# Harnessing the endophytic *Clavispora lusitaniae* for the biogenic synthesis of ethylene glycol-capped cerium oxide nanoparticles: a green approach to combat respiratory *Pseudomonas aeruginosa*

**DOI:** 10.1186/s12934-026-03091-x

**Published:** 2026-08-20

**Authors:** Hoda S. Nouh, Nessma A. El-Zawawy, Shimaa El-Sapagh, Ebrahim M. Shalamesh, Sally M. Metwally

**Affiliations:** 1 Botany and Microbiology Department, Faculty of Science, Tanta University, Tanta, 31511 Egypt; 2 Institute for Integrative Biology of the Cell (I2BC), CEA, CNRS, Université Paris-Saclay, 91198 Gif-sur-Yvette, France

**Keywords:** *Artemisia judaica*, Endophytic yeast, *Clavispora lusitaniae*, CeO_2_NPs, Antibacterial, Biofilm, Virulence genes

## Abstract

**Background:**

The utilization of endophytes as specialized microbial cell factories offers a sustainable and high-efficiency platform for the biosynthesis of functionalized nanomaterials. This study investigates the potential of a novel endophytic yeast, *Clavispora lusitaniae*, to produce cerium oxide nanoparticles (CeO₂NPs) and evaluates their efficacy against extensively drug-resistant (XDR) *P. aeruginosa*.

**Results:**

An endophytic yeast, *C. lusitaniae*, was isolated for the first time from the medicinal plant Artemisia judaica and employed for the biosynthesis of CeO₂NPs, followed by surface capping with ethylene glycol (EG) to modify the nanoparticle surface properties. The synthesized nanoparticles were characterized using UV-Vis, FTIR, XRD, TEM, and DLS. These analyses confirmed the formation of spherical EG-CeO₂NPs with an average size of 8–20 nm. EG-CeO₂NPs exhibited strong antibacterial activity against XDR *P. aeruginosa* strains, with a minimum inhibitory concentration ranging from 0.6 to 1.25 mg/mL. Furthermore, these nanoparticles demonstrated potent anti-biofilm efficacy, achieving reductions of up to 89%. Mechanistic investigations demonstrated that EG-CeO₂NPs disrupt bacterial cell membranes, leading to intracellular protein leakage and elevated lipid peroxidation (indicated by increased malondialdehyde levels). Furthermore, the expression levels of quorum sensing (e.g., lasR and rhlR) and virulence-associated genes (e.g., toxA and exoS) were markedly downregulated by up to 87% compared to untreated controls.

**Conclusions:**

This study establishes *C. lusitaniae* as a robust microbial cell factory for synthesizing functionalized CeO₂NPs with potent activity against XDR *P. aeruginosa*. Our results demonstrate the potential of microbial bioprocessing in engineering prospective nanotechnological platforms to combat antimicrobial resistance. However, further studies evaluating colloidal behavior under physiologically relevant conditions, mammalian-cell cytotoxicity, and in vivo efficacy are required before biomedical translation.

**Supplementary Information:**

The online version contains supplementary material available at 10.1186/s12934-026-03091-x.

## Background

Currently, antimicrobial resistance (AMR) is categorized as a major global health threat, posing a critical risk to modern medicine and public safety worldwide [1]. Globally, infections caused by antibiotic-resistant bacteria are responsible for approximately 2000 deaths per day. The prevalence of resistance to conventional antibiotics is projected to reach 40–60% by 2030, with annual mortality rates potentially rising to 10 million deaths by 2050, particularly in Asia and Africa [2]. The Gram-negative opportunist *P. aeruginosa* is characterized by its ubiquitous nature and its significant role in manifesting a broad array of acute and long-term human infections [3]. It is associated with severe and potentially fatal diseases, with mortality rates reaching up to 40% [4–6]. Over the past decades, antimicrobial resistance in *P. aeruginosa* clinical isolates has increased dramatically [7–9]. Of particular concern is the emergence of extensively drug-resistant (XDR) strains, which significantly complicate treatment options and contribute to increased morbidity and mortality [10, 11].

*P. aeruginosa* respiratory infections are particularly lethal in patients with cystic fibrosis or chronic obstructive pulmonary disease. The pathogen forms recalcitrant biofilms governed by complex QS networks, making traditional antibiotics largely ineffective [12–17]. Such obstacles underscore the necessity of developing unconventional pharmacological approaches beyond traditional antibiotics [18].

To address these challenges, the development of innovative biotechnological platforms has become a priority. The field of nanotechnology has emerged as a promising strategy, where nanoparticles (NPs) exhibit unique physicochemical properties that bypass conventional resistance mechanisms [19]. Among them, cerium oxide nanoparticles (CeO₂NPs) have attracted considerable attention due to their redox-active surface, oxygen vacancy defects, and ability to switch between Ce³⁺ and Ce⁴⁺ states, which contribute to their antimicrobial and antibiofilm properties [20, 21]. However, the transition toward sustainable and “green” production methods has shifted the focus toward microbial cell factories as a superior alternative to traditional chemical synthesis [22].

While various microbial systems, including fungi such as *Aspergillus* and *Fusarium* spp., or bacteria, have been explored for the green synthesis of metal oxide nanoparticles, the biogenic production of CeO₂ NPs remains relatively limited compared to silver or gold nanostructures [23, 24]. Previous fungal-mediated CeO₂ synthesis studies often faced challenges regarding particle size control and long-term colloidal stability in physiological media [25, 26]. Endophytic yeasts have recently emerged as a sophisticated and underexplored bioprocessing platform. Residing asymptomatically within plant tissues, they constitute a rich source of extracellular proteins, enzymes, polysaccharides, and other bioactive metabolites that can simultaneously mediate metal ion reduction and nanoparticle stabilization [27–30]. Due to these distinct physiological characteristics, they have attracted increasing attention as sustainable biocatalysts for the fabrication of metallic and metal oxide nanostructures [21, 31]. *Artemisia judaica*, a medicinal plant known for its diverse pharmacological properties [32], harbors a unique endophytic microflora with untapped biotechnological potential [33]. Despite this potential, the utilization of endophytic yeasts from *A. judaica* specifically as cell factories for the biogenic production of CeO₂ NPs has not been previously established. Various studies have reported the biosynthesis of CeO₂ nanoparticles using bacteria or filamentous fungi. However, to the best of our knowledge, this is the first study to employ the endophytic yeast *C. lusitaniae* for this purpose. Moreover, the use of *C. lusitaniae* isolated from *Artemisia judaica* as a microbial cell factory for the production of EG-capped CeO₂ nanoparticles has not been previously reported. This knowledge gap highlights the novelty of the present work and distinguishes it from previously published microbial-mediated nanoceria studies.

In addition to green biosynthesis, surface capping is a pivotal strategy to maximize nanoceria stability, biocompatibility, and targeted therapeutic efficacy [34, 35]. Surface capping agents regulate crystal growth, reduce excessive aggregation, improve colloidal behavior, and ultimately influence the biological performance of nanoparticles. These improvements may enhance nanoparticle dispersion in biological environments, increase the accessibility of reactive surface sites, and consequently improve antimicrobial performance [36]. Ethylene glycol (EG), owing to its hydroxyl-rich structure, has been widely employed as a structure-directing and capping agent during nanoparticle synthesis. Nevertheless, its application as a surface capping molecule for biologically synthesized CeO₂ nanoparticles remains poorly explored, particularly in microbial cell factory-based systems [37, 38].

Based on the currently available literature, no published work has integrated endophytic yeast-mediated biosynthesis of CeO₂ nanoparticles with EG surface capping and subsequent mechanistic evaluation of antibacterial, antibiofilm, and quorum sensing inhibitory activities against extensively drug-resistant respiratory *P. aeruginosa*. This integrated strategy distinguishes the present work from previously reported CeO₂ nanoparticle studies by combining an endophytic yeast-based microbial cell factory, EG surface engineering, and mechanistic antibacterial evaluation against respiratory XDR *P. aeruginosa*.

Therefore, the present study reports, for the first time, a biotechnological platform employing the endophytic yeast *C. lusitaniae* isolated from *Artemisia judaica* as a microbial cell factory for the biosynthesis of EG-capped CeO₂ nanoparticles. Furthermore, the antibacterial and antibiofilm activities of the synthesized nanoparticles were evaluated against extensively drug-resistant (XDR) *P. aeruginosa* strains isolated from cystic fibrosis patients. By integrating microbial synthesis with surface engineering, this study investigates the impact of the produced nanostructures on quorum sensing-regulated pathways and biofilm-associated mechanisms, aiming to explore the potential of this microbial cell factory-based approach for combating persistent respiratory infections.

## Materials and methods

### Plant collection

Fresh aerial parts of the wild medicinal plant *Artemisia judaica* L. were collected from the Salouga and Ghazal Islands Natural Reserve, located north of Aswan City, southern Egypt (24° 05ʹ N, 32° 53ʹ E), as shown in Fig. S1. Taxonomic authentication of the botanical species was conducted by Prof. Dr. Kamal Shaltout (Department of Botany, Faculty of Science, Tanta University, Egypt) [39]. A voucher specimen (No. 304-303) was archived at the Tanta University Herbarium (TUH), Faculty of Science, Egypt. The harvested plant tissues were placed in sterilized, airtight containers and transported to the laboratory facility under refrigerated conditions (4 °C).

### Isolation and identification of endophytic yeast

An endophytic yeast strain, designated as SH.1, was isolated from healthy leaves of *Artemisia judaica* L. The isolation procedure was performed according to El-Zawawy et al. [40] with minor technical adjustments. Briefly, collected leaf samples were thoroughly washed under running tap water to remove adhering debris. Surface sterilization was carried out by sequential immersion in sterile distilled water (1 min), sodium hypochlorite solution (2.5%, 4 min), and 70% ethanol (30 s), followed by rinsing in sterile distilled water (2 min). The efficacy of the sterilization was validated through the inoculation of the terminal rinse water onto nutrient agar (NA) media for bacteria and Sabouraud dextrose agar (SDA) for fungi. The absence of microbial growth indicated successful surface sterilization. Sterilized leaf segments (approximately 4 mm each; five segments per plate) were aseptically placed onto SDA (Oxoid) supplemented with chloramphenicol and incubated at 30 °C for 48 h. Emerging yeast colonies originating from internal tissues. Distinct colonies were subcultured onto fresh SDA plates and purified on selective medium (CHROMagar *Candida*, Oxoid).

Preliminary identification was performed based on morphological characteristics and scanning electron microscopy (SEM), following standard protocols [41, 42]. Molecular characterization involved sequencing the internal transcribed spacer (ITS) domain. The obtained ITS sequence of isolate SH-1 was compared with reference sequences retrieved from the NCBI GenBank database using BLASTn. Multiple sequence alignment was performed using MEGA version 12.1.2 [43]. Phylogenetic reconstruction was carried out using the Maximum Likelihood (ML) method based on the Tamura–Nei nucleotide substitution model [44]. The robustness of the inferred topology was evaluated using 1000 bootstrap replicates. *Saccharomyces cerevisiae* CBS 1171 (NR_111007) was included as the outgroup. The resulting phylogenetic tree confirmed the taxonomic position of isolate SH-1 within the genus *Clavispora*.

### Endophytic yeast (SH.1) mediated synthesis of CeO₂NPs

The endophytic yeast strain SH-1 was utilized for the biological production of CeO₂NPs. Five distinct yeast colonies were introduced into 500 mL of Sabouraud dextrose broth (SDB) and incubated at 30 °C for 48 h with continuous agitation until reaching a standardized stationary phase (OD₆₀₀ = 1.5, equivalent to ~ 12.5 g/L wet biomass). The medium was then centrifuged at 12,000 rpm for 10 min, and the cell-free supernatant was harvested and stored at 4 °C. CeO₂NPs were synthesized via a modified solution combustion approach [24]. Briefly, cerium nitrate hexahydrate (20 mmol; Loba Chemie, India) was dissolved in 100 mL of distilled water and mixed with 20 mL of the cell-free filtrate. This mixture was stirred at 80 °C for 4 to 6 h. The formation of nanoparticles was evidenced by the transition of the white precipitate to a yellowish-brown color. The obtained solid was recovered and calcined at 400 °C for 2 h to yield CeO₂NPs in nanopowder form.

### In silico safety evaluation of ethylene glycol as a capping agent for CeO₂NPs

Ethylene glycol (EG) was utilized as a surface-stabilizing agent during post-synthesis modification of CeO₂NPs to improve their colloidal stability and dispersion characteristics. Given its direct interaction with the nanoparticle surface, an assessment of its predicted pharmacokinetic behavior and toxicity profile is essential to ensure preliminary information regarding the safety of EG-capped nanoparticles for potential biomedical applications. The absorption, distribution, metabolism, and excretion (ADME) properties of EG were predicted using the SwissADME web server (http://www.swissadme.ch), while toxicity endpoints were assessed using the ProTox-III platform (https://tox.charite.de/protox3/). These complementary computational analyses provided an initial in silico safety assessment of the capping agent.

### Ethylene glycol capping and characterization of biosynthesized EG-CeO_2_NPs

For surface modification, the biosynthesized CeO₂NPs were re-dispersed in deionized water, followed by the gradual addition of EG at a 1:1 (v/v) ratio under continuous stirring. The mixture was maintained at 60 °C for 2 h to facilitate the adsorption of ethylene glycol molecules onto the nanoparticle surface, thereby improving surface functionalization [45]. The resulting ethylene glycol-capped cerium oxide nanoparticles (EG-CeO₂NPs) were collected by centrifugation (3000 rpm, 5 min), washed thrice with deionized water, and then twice with absolute ethanol to remove unbound molecules. Finally, the precipitate was dried overnight at 65 °C.

Biosynthesized EG-CeO₂NPs were characterized using multiple analytical techniques. UV–visible spectroscopy (Shimadzu UV-1800, Japan) was performed in the wavelength range of 200–800 nm with a resolution of 1 nm. Fourier transform infrared (FTIR) analysis was carried out using a Vertex 70-RAM II Bruker spectrometer (Bruker Analytical GmbH, Germany) over a spectral range of 400–4000 cm⁻¹ to identify functional groups involved in nanoparticle stabilization and capping. The crystalline structure of the nanoparticles was analyzed by X-ray diffraction (XRD) using an XPERT-PRO powder diffractometer (PANalytical, Netherlands) over a 2θ range of 20°–80°, with a minimum step size of 0.001° and Cu Kα radiation (λ = 1.5406 Å). The average crystallite size (*D*) of the biosynthesized EG-CeO₂NPs was calculated using the Debye–Scherrer equation: D = K *λ*\*β* cos *θ* where K is the shape factor (0.9), *λ* is the X-ray wavelength (1.5406 Å), *θ* is the Bragg diffraction angle, and *β* is the full width at half maximum (FWHM) of the most intense diffraction peak (111) in radians. Morphological assessment was conducted via high-resolution transmission electron microscopy (HR-TEM, JEOL JEM-2100, 200 kV). For TEM analysis, Specimen preparation involved depositing a droplet of the colloidal suspension onto a 300-mesh copper grid coated with Formvar carbon. Following air-drying at room temperature, the size uniformity and polydispersity index (PDI) were evaluated. Particle size distribution and polydispersity index (PDI) were determined using dynamic light scattering (DLS) (Zetasizer Nano ZN, Malvern Panalytical Ltd., UK) analysis was executed at a constant angle of 173° and a thermal setting of 25 °C, with all trials conducted in triplicate to ensure reproducibility. Furthermore, the zeta potential was quantified using the same system to assess the electrostatic stability of the particles.

### Bacterial strains and culture conditions

A standard strain of *P. aeruginosa* (PA) 27853 from the American Type Culture Collection (ATCC), together with six XDR and strong biofilm producing strains of *P. aeruginosa* (PA1–PA6) that had previously been collected from infected human lungs were used [46]. Bacterial isolates were cryopreserved at − 80 °C in Luria broth (LB) supplemented with a 25% (v/v) glycerol solution. For experimental reactivation, the strains were subcultured onto LB agar medium and maintained at 37 °C for an 18-hour incubation period. To obtain an active overnight culture, one colony was introduced into 30 mL of LB and incubated for 18 h at 37 °C with agitation at 130 rpm.

### Antibacterial activity

The antibacterial potential of EG-CeO₂NPs against the designated strains was evaluated via the agar well diffusion technique, following a modified version of established protocols [47, 48]. Briefly, agar plates were inoculated with 100 µL of bacterial broth adjusted to a density of 10⁶ CFU/mL to accommodate the slower agar diffusion kinetics of biogenic nanoparticles, thereby optimizing sensitivity for precise sub-MIC determination in subsequent investigations. Using a sterile pipette, fifty microliters of EG-CeO₂NPs (pre-dissolved in sterile distilled water) was introduced into 5-mm diameter wells at concentrations of 2.5, 5, and 10 mg/mL. The minimum inhibitory concentration (MIC) was determined using a resazurin-based microdilution assay [49]. Microbial viability was assessed by monitoring the transition of resazurin dye from dark blue to pink, with the latter indicating metabolic activity. The MIC was identified as the threshold concentration that completely precluded this color shift, signifying total growth suppression. Determination of the minimum bactericidal concentration (MBC) was executed by transferring aliquots from the experimental wells onto LB agar plates, followed by 24-h incubation. The MBC was identified as the threshold concentration capable of achieving a 99.5% decline in the initial inoculum [50]. Streptomycin (200 µg/mL) served as the positive reference and EG alone (10 mg/mL) was included as an additional control to evaluate the possible contribution of the capping agent to the observed antibacterial activity. All experiments were conducted in triplicate to ensure reproducibility. Sub-MIC values were then identified for subsequent mechanistic studies. In addition, the impact of EG-CeO₂NPs at MIC and 1/2 x MIC concentrations on structural alterations in bacterial cells were visualized via transmission electron microscopy (TEM) utilizing a JEOL JEM-1400 (JAPAN) system at the National research center, Cairo, Egypt.

### Growth curve at sub‑MICs of EG-CeO₂NPs

One hundred microliters of overnight culture suspensions containing 1 × 10^6^ CFU/mL of bacterial strains were combined with an equal volume of EG-CeO₂NPs dissolved in distilled water. This resulted in concentrations of 1/2, 1/4, 1/8, and 1/16 times the MICs in Mueller-Hinton broth. The mixtures were then subjected to incubation at 37 °C for a duration of 24 h within a rotary shaking incubator maintained at 120 rpm. The turbidity of the bacterial cultures (measured at OD660 nm) was evaluated after 24 h [51]. Bacteria cultivated in wells that did not contain EG-CeO₂NPs were served as positive controls.

### Lipid peroxidation (LPO) assay and protein leakage

The induction of oxidative stress and subsequent lipid peroxidation were evaluated by quantifying malondialdehyde (MDA) levels using the thiobarbituric acid (TBA) assay [52, 53]. Briefly, 1 mL of the treated bacterial suspension was precipitated with 2 mL of 10% trichloroacetic acid. Subsequently, 4 mL of 0.67% TBA reagent was added, and the mixture was heated at 95 °C for 20 min to catalyze the formation of the MDA-TBA chromogenic adduct. Following rapid cooling to ambient temperature, the samples were centrifuged at 6000 rpm for 15 min. The resulting supernatant was analyzed spectrophotometrically, with the absorbance recorded at 532 nm to determine the extent of membrane damage.

Intracellular protein leakage was quantified as a direct indicator of cytoplasmic membrane compromise in the treated microorganisms [54, 55]. To evaluate this, bacterial suspension was incubated with EG-CeO₂NPs at a sub-inhibitory concentration (½ × MIC). Following treatment, the cell-free supernatants were collected and 1 mL aliquots were reacted with an equal volume of Coomassie Brilliant Blue (CBB-250) reagent. The mixture was incubated in darkness at ambient temperature for 5 min to facilitate protein-dye binding. Subsequently, the protein concentration was determined spectrophotometrically by measuring the absorbance at 595 nm using a microplate reader [56, 57].

### Effect of EG-CeO₂NPs on biofilm formation

The inhibitory efficacy of EG-CeO₂NPs on biofilm development was evaluated via the crystal violet (CV) staining assay [41, 58]. Bacterial inocula (5 × 10⁶ CFU/mL) were formulated in Trypticase soy broth (TSB) enriched with 0.5% glucose. Aliquots (100 µL) were co-incubated with equivalent volumes of EG-CeO₂NPs at diverse sub-inhibitory concentrations (1/2, 1/4, 1/8, and 1/16 × MIC) in 96-well microtiter plates. Following 24 h of incubation at 37 °C, planktonic cells were gently removed by washing with phosphate-buffered saline (PBS, pH 7.0) three times. Adherent biofilms were air-dried for 15 min and heat-fixed at 60 °C for 30 min, followed by staining with 0.1% crystal violet for 15 min. Excess dye was removed by rinsing with PBS, and the biofilm-associated crystal violet was solubilized in 96% ethanol for 20 min. Biofilm biomass was assessed spectrophotometrically at 595 nm. Biofilm inhibition (%) was normalized relative to the untreated control using the following equation:

$${\mathrm{Biofilm}}\;{\mathrm{inhibition}}/{\mathrm{eradication}}\left( \% \right)=[({\mathrm{ODcontrol}} - {\mathrm{ODtreated}})/{\mathrm{ODcontrol}}] \times {\mathrm{1}}00.$$

The data points represent the mean values with standard deviations from three different experiments performed in triplicate. Samples were analyzed with a field emission scanning electron microscope (FE-SEM, Quanta FEG 250, FEI Company, USA) at the Electron Microscopy Unit, National Research Center, Cairo, Egypt, to observe the morphological changes in the biofilm structure.

### Effect of EG-CeO₂NPs on preformed biofilm

The eradication efficacy of EG-CeO₂NPs against established biofilms was investigated using the crystal violet assay. Following biofilm maturation for 24 h, the supernatant containing planktonic cells was aspirated, and the wells were gently rinsed with 1× PBS. Mature biofilms were then treated with 200 µL of EG-CeO₂NPs at 1×, 2×, and 4× MIC, while control wells received an equal volume of fresh broth. After a second incubation period (24 h at 37 °C), the remaining biofilm biomass was quantified via CV staining. Data are expressed as the mean ± standard deviation (SD) of three independent experiments performed in triplicate.

### Quantitative real-time PCR analysis of biofilm and virulence-related genes in *P. aeruginosa*

To clarify the molecular effects of EG-CeO₂NPs on the transcriptional patterns of biofilm- and virulence-associated genes in *P. aeruginosa* (strain PA-5), quantitative real-time PCR (qRT-PCR) was performed. Total RNA was extracted from both nanoparticle-treated and control cultures utilizing the RNeasy Mini Kit (Qiagen NV, Netherlands) in strict accordance with the manufacturer’s protocol. The yield and integrity of RNA were evaluated using a NanoDrop 2000c spectrophotometer (Thermo Fisher Scientific, USA). Subsequently, 1 µg of pure RNA was reverse-transcribed into complementary DNA (cDNA) utilizing the High-Capacity cDNA Reverse Transcription Kit (Applied Biosystems).

The expression levels of 14 targeted genes including regulatory (*lasR/I*, *rhlR/I*, *pqsR/*), biofilm-associated (*pelF*, *pslA*, *algD*), and effector virulence factors (*lasB*, *plcH*, *toxA*, *exoS*, *aprA*, gacA) were quantified using the ViiA™ 7 Real-Time PCR System. Primer sequences are provided in Supplementary Table S1 [59, 60]. Amplification was executed using a three-step thermal cycling profile: an initial denaturation at 95 °C for 3 min; followed by 45 cycles of denaturation at 95 °C for 15 s, annealing at 68 °C for 30 s, and extension at 72 °C for 30 s. Following amplification, a continuous melting curve analysis was conducted (50 °C to 99 °C, 5 s hold per step) in the green channel to verify amplicon specificity and eliminate primer-dimer artifacts. Data normalization was performed using the *proC* housekeeping gene as an internal reference to determine relative fold-change expression. All qRT-PCR assays were conducted in triplicate using three independent bacterial cultures to ensure reproducibility.

### Statistical analysis

All experimental trials were performed using a single batch of synthesized nanoparticles, and the reported replicates represent repeated measurements from this same nanoparticle preparation to ensure consistency. Data are presented as mean ± standard deviation (SD). Prior to statistical evaluation, data normality was assessed using the Shapiro-Wilk test, and variance homogeneity was verified. Statistical analyses were performed using GraphPad Prism version 11.0.2 (GraphPad Software, San Diego, CA, USA). Comparisons among multiple groups were carried out using one-way or two-way analysis of variance (ANOVA), as appropriate, followed by Dunnett’s or Tukey’s multiple comparisons tests, with the corresponding untreated control used as the reference group. Exact *P* values are reported alongside standard significance categories where appropriate. Statistical significance was considered at *P* < 0.05.

## Results and discussion

### Isolation and identification of the endophytic yeast strain (SH.1)

**Fig. 1:**
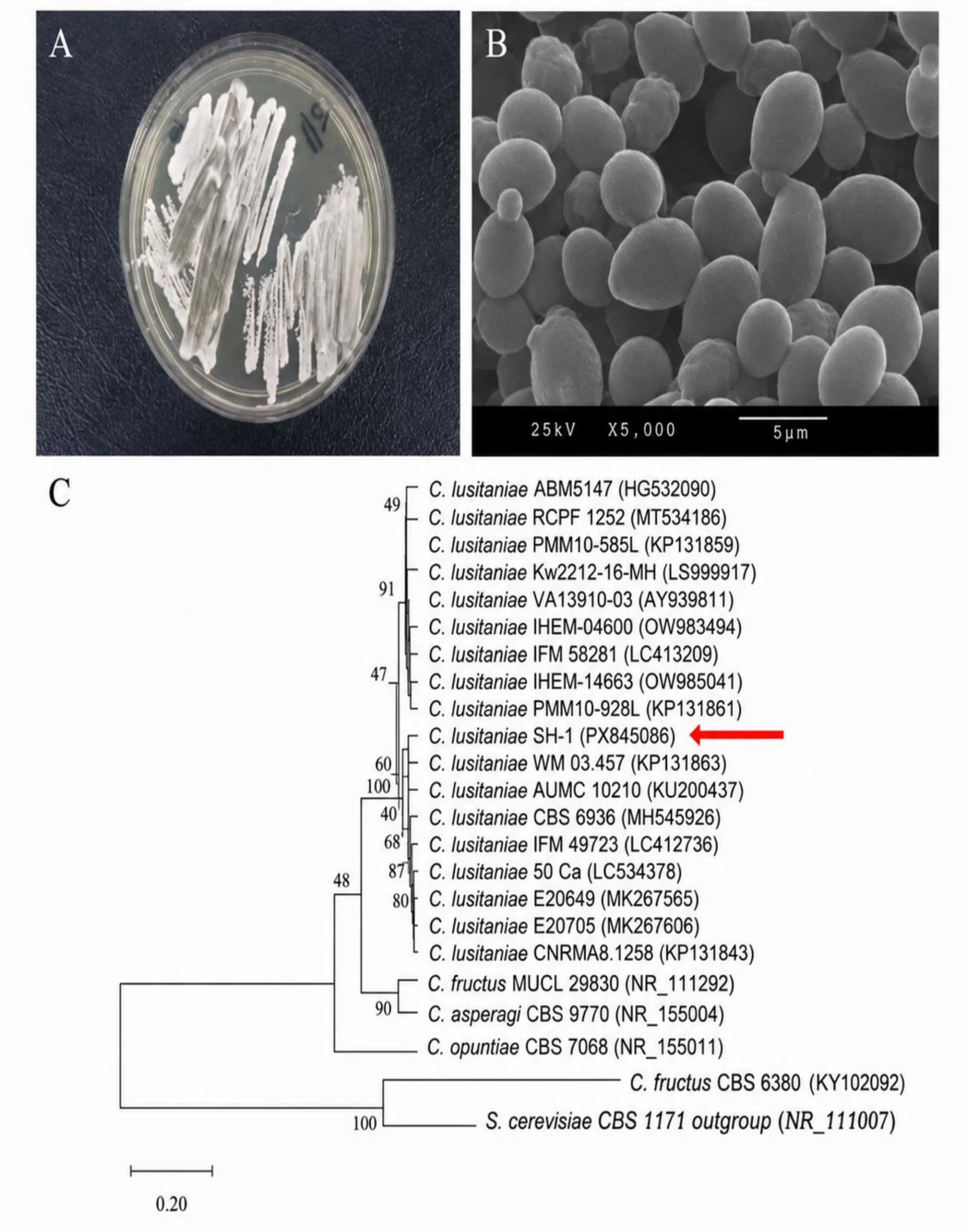
Identification of endophytic yeast *C. lusitaniae SH-1.* **A** Colony morphology on Sabouraud dextrose agar. **B** Scanning electron micrograph showing budding yeast cells. **C** ITS-rDNA Maximum Likelihood phylogenetic tree showing the position of SH-1 (PX845086, red arrow) among related taxa, with *S. cerevisiae* CBS 1171 (NR_111007) as outgroup

The endophytic yeast isolate SH.1 was successfully recovered from surface-sterilized leaves of *A. judaica* L (Fig. S1). following incubation on SDA at 30 °C for 48 h. Macroscopically, the colonies exhibited a creamy-white coloration with a smooth morphology and entire margins (Fig. 1A), which are typical characteristics of yeast species. Microscopic examination using electron microscopy revealed predominantly oval-shaped cells with smooth surfaces, along with actively budding cells (Fig. 1B), indicating yeast proliferation.

Based on these morphological features, the isolate was tentatively assigned to yeast taxa. To achieve precise identification, morphological identification was further confirmed by ITS sequence analysis. Phylogenetic analysis based on the Maximum Likelihood method demonstrated that isolate SH-1 clustered within the *C. lusitaniae* lineage and was clearly separated from closely related *Clavispora* species. The obtained ITS sequence was deposited in the GenBank database under accession number PX845086, confirming the taxonomic identity of the isolate as *C. lusitaniae* (Fig. 1C).

Interestingly, the isolation of *C. lusitaniae* as an endophyte from *A. judaica* is reported here for the first time. This finding contrasts with the study by Menaa et al. [61], who isolated actinobacteria from *A. judaica* L. in Algerian desert regions. This evidence supports the concept that the colonization of plants by endophytic fungi is not solely dependent on host specificity; environmental conditions may also play a critical role [62]. As previously stated, environmental factors (e.g., nutrient and temperature) will influence the diversity of endophytes that inhabit plants. Therefore, the successful identification of *C. lusitaniae* suggests its potential as a valuable microbial resource for the eco-friendly and sustainable production of CeO_2_NPs.

### Pharmacokinetic and safety profile of ethylene glycol for capping CeO_2_NPs

The pharmacokinetic behavior and predicted toxicological profile of EG (Fig. S1) were evaluated using complementary in silico platforms. The ADMET analysis focused on EG as the primary interfacial layer interacting with biological systems. Since current computational tools are optimized for small organic molecules and lack parameters for inorganic nanoparticles, evaluating the capping agent’s safety is a necessary preliminary step in assessing the overall biocompatibility of the functionalized nanoplatform.

**Fig. 2:**
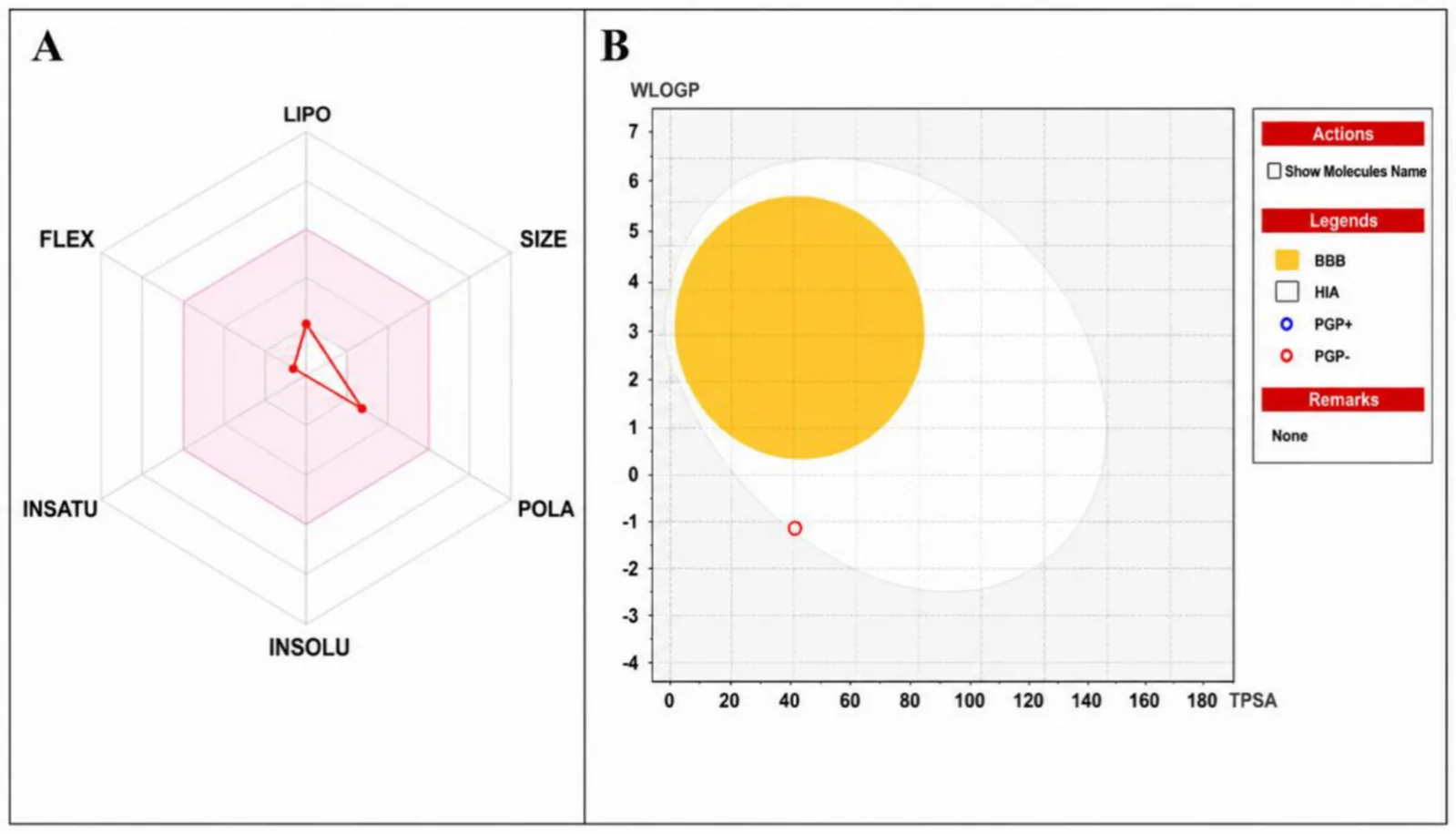
Computational ADMET profiling of ethylene glycol (EG). **A** Bioavailability radar showing drug-likeness properties. **B** BOILED-Egg model indicating high GI absorption potential and absence of CNS permeation

The canonical SMILES notation and molecular formula of EG are presented in Table S2, while its physicochemical properties are summarized in Table S2. EG exhibited a low molecular weight (62.07 g/mol), a topological polar surface area (TPSA) of 40.46 Å², two hydrogen-bond donors, two hydrogen-bond acceptors, and a fully saturated carbon skeleton (fraction C_sp3_ = 1.00). Collectively, these properties indicate a small, highly polar molecule favorable for nanoparticle surface functionalization. Lipophilicity analysis (Table S2) revealed a consensus Log_*P*_ value of − 0.70, indicating pronounced hydrophilicity. Such a low partition coefficient is advantageous for maintaining nanoparticle dispersion in aqueous biological environments and may contribute to improved colloidal behavior following surface capping. Consistent with these findings, the SwissADME bioavailability radar (Fig. 2A) demonstrated that EG falls within the recommended ranges for molecular size, polarity, flexibility, and solubility.

The BOILED-Egg model (Fig. 2B) predicted high gastrointestinal absorption while indicating limited passive permeation across the blood–brain barrier (BBB). These pharmacokinetic predictions are supported by drug-likeness analysis (Table S2, where EG satisfied the Lipinski, Veber, and Egan criteria without violations. Although violations of the Ghose and Muegge filters were observed, these reflect the small molecular size and simple structure of EG rather than unfavorable physicochemical properties. Water solubility prediction (Table S2) consistently classified EG as highly soluble across the ESOL, Ali, and Silicos-IT models, confirming excellent aqueous solubility, which is beneficial for homogeneous nanoparticle dispersion and may facilitate interactions between the nanoparticle surface and biological media.

The toxicity profile predicted by ProTox-III (Table S3) suggested ‘Inactive’ probabilities for hepatotoxicity, mutagenicity, and immunotoxicity, whereas moderate probabilities were predicted for cardiotoxicity and a BBB-related toxicity endpoint. These findings do not contradict the SwissADME predictions, as the two computational platforms evaluate distinct biological characteristics. SwissADME predicts passive pharmacokinetic properties (e.g., absorption and permeation), whereas ProTox-III estimates potential toxicity endpoints using independent machine-learning algorithms. Consequently, the limited BBB permeation predicted by SwissADME does not conflict with the BBB-related toxicity alert from ProTox-III, as the latter reflects a predicted toxicological endpoint rather than actual permeability.

However, a critical toxicological caveat concerns nephrotoxicity. Systemically, EG metabolizes into oxalic acid, a potent inducer of acute kidney injury. Although the server predicted an ‘Inactive’ status, the marginal probability score of 0.55 resides near random chance, indicating low computational confidence. Delivering an EG-functionalized platform directly into compromised respiratory tissues (e.g., in cystic fibrosis) introduces serious toxicological concerns that cannot be dismissed by in silico data alone. Consequently, the computational predictions should not be interpreted as evidence of overall safety, but rather as preliminary indicators requiring experimental validation.

Overall, these complementary computational analyses provide a preliminary assessment of the physicochemical behavior and safety profile of EG as an organic capping agent. However, these predictions apply only to the free EG molecule and should not be extrapolated to the biological behavior of the complete EG-CeO₂NP formulation. Therefore, additional experimental studies are essential to comprehensively evaluate the safety of the final nanoparticle system prior to potential biomedical applications.

### Characterization of biosynthesized EG-CeO_2_NPs

Conventional nitrate-assisted synthesis of CeO_2_NPs is often associated with limitations such as particle aggregation and high polydispersity [25], which may negatively affect their physicochemical properties and biological performance. These challenges emphasize the need for alternative, biologically driven synthesis strategies.

The present study demonstrated, for the first time, the use of *C. lusitaniae* as a biogenic system for the synthesis of CeO₂NPs followed by post-synthetic surface functionalization with EG. The involvement of microbial metabolites in the synthesis process may contribute to both the reduction of metal precursors and the stabilization of the formed nanoparticles. Furthermore, ethylene glycol capping is expected to enhance nanoparticle stability and dispersibility by minimizing aggregation and controlling particle growth, as previously reported for polyol-based capping systems [38, 63]. This integration of biological synthesis and surface modification yield nanoparticles with improved physicochemical characteristics and enhanced biological functionality [64].

### UV–Visible spectroscopy

**Fig. 3:**
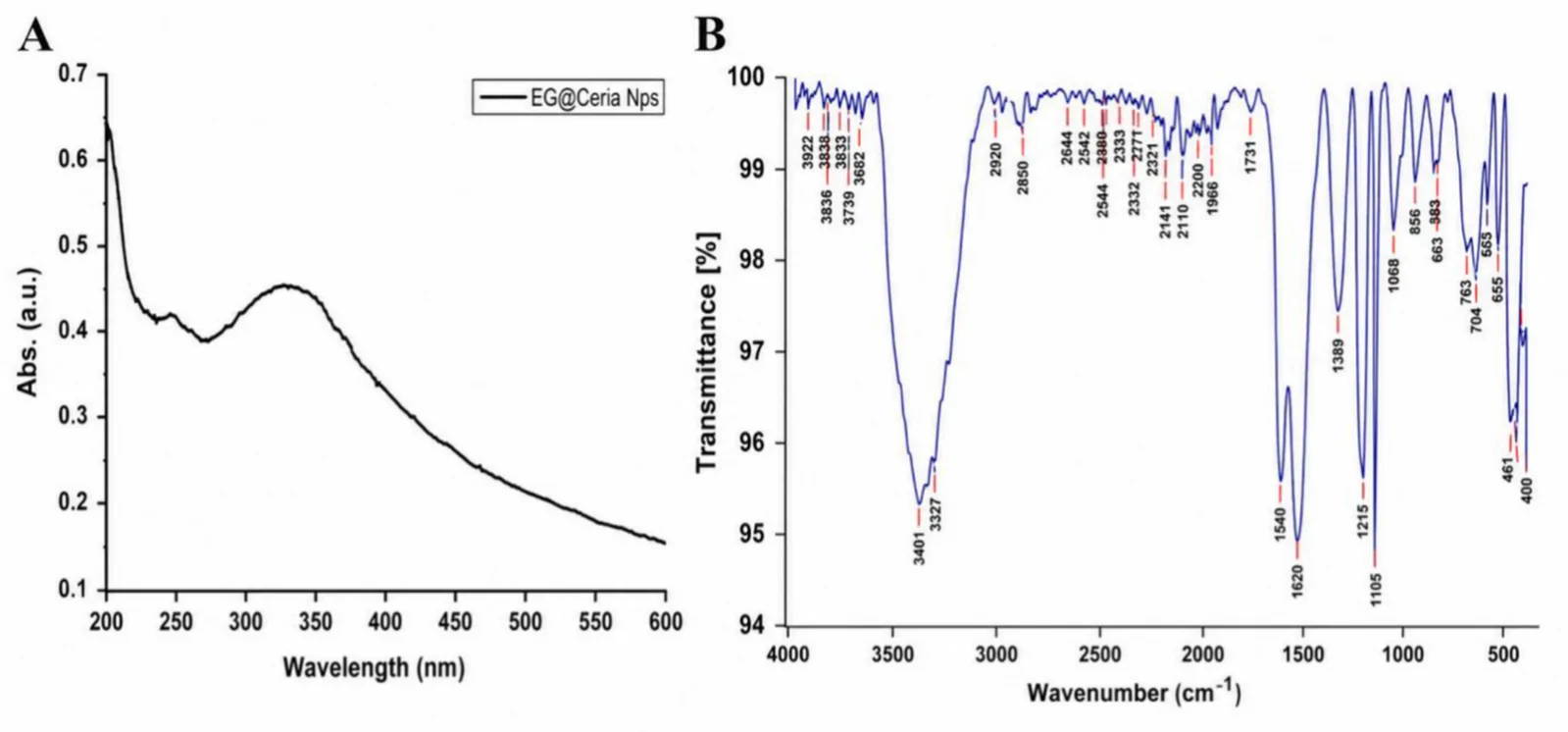
UV-vis spectrum (**A**), and FTIR spectrum (**B**) of biosynthesized EG-CeO_2_NPs

The UV–visible absorption spectrum of the biosynthesized EG-CeO₂NPs exhibited a characteristic absorption band in the UV region, with a noticeable shoulder around 300–330 nm (Fig. 3A), attributed to the O²⁻ → Ce⁴⁺ charge transfer transition. This feature confirms the successful formation of CeO_2_NPs. It is well established that Ce³⁺ ions exhibit absorption between 230 and 260 nm, whereas Ce⁴⁺ ions absorb in 300–400 nm [65]. In nanoceria systems, cerium predominantly exists in the oxidized Ce⁴⁺ state; however, a reduction in particle size is often accompanied by the generation of oxygen vacancies, leading to partial conversion of Ce⁴⁺ to Ce³⁺ at the nanoparticle surface. This mixed valence state (Ce³⁺/Ce⁴⁺) is a hallmark feature of CeO_2_NPs, playing a crucial role in their redox behavior and biological activity. The observed spectral features agree with previous reports [66], supporting the successful synthesis of nanoscale CeO with characteristic electronic transitions.

### Fourier-transform infrared spectroscopy

FTIR spectrum of the biosynthesized EG-CeO₂NPs confirmed the successful formation and surface functionalization of the nanoparticles, consistent with previous studies [25, 67, 68]. FTIR analysis revealed the dual-functionalization of the nanoparticles by both microbial metabolites and the EG capping agent (Fig. 3B). A broad absorption band centered around 3400 cm⁻¹ was attributed to O–H stretching vibrations originating from hydroxyl groups of yeast-derived biomolecules and EG. The characteristic bands at 2950 and 2850 cm⁻¹ correspond to the asymmetric and symmetric stretching vibrations of CH₂ groups, confirming successful EG capping. Bands observed at 1640 and 1556 cm⁻¹ were assigned to amide I/amide II vibrations, alongside adsorbed water molecules, suggesting the participation of microbial proteins in nanoparticle stabilization. Furthermore, the bands at 1215, 1156, and 1068 cm⁻¹ correspond to C–O stretching vibrations of EG adsorbed onto the nanoparticle surface, while the absorption band below 700 cm⁻¹ was assigned to Ce–O lattice vibrations, confirming the formation of crystalline CeO_2_NPs.

### Transmission electron microscopy (TEM) and X-ray diffraction (XRD)

**Fig. 4:**
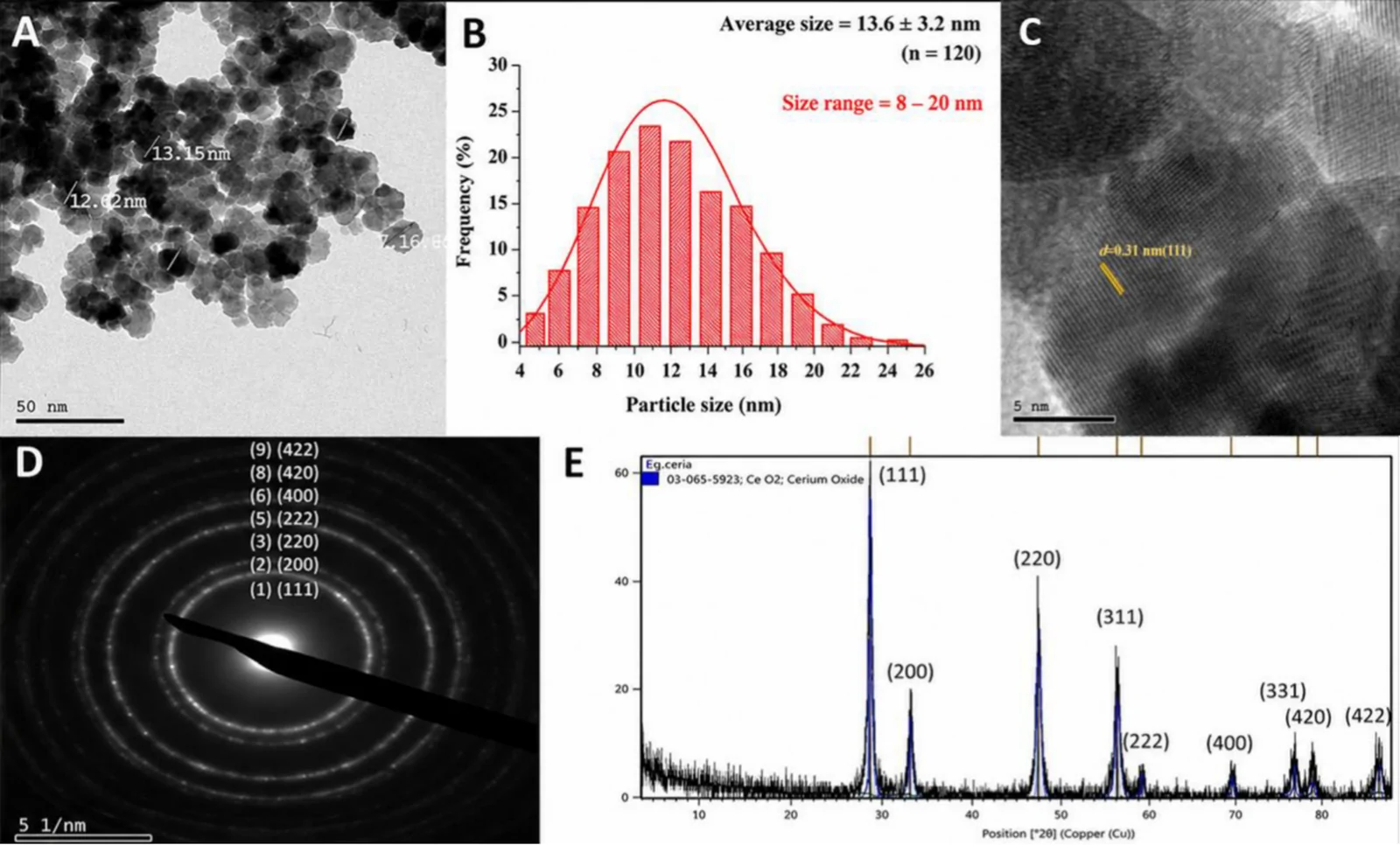
Characterization of biosynthesized EG-CeO2 NPs. **A** TEM micrograph showing spherical morphology. **B** Size distribution histogram (13.6 ± 3.2 nm, *n* = 120). **C** HRTEM showing lattice fringes (d-spacing ~ 0.31 nm, (111) plane). **D** SAED pattern (polycrystalline). **E** XRD pattern indexed to cubic fluorite CeO_2_ (JCPDS No. 03-065-5923)

TEM analysis revealed that the biosynthesized EG-CeO₂NPs were predominantly spherical, with an average particle size of 13.6 ± 3.2 nm (*n* = 120) and a range of 8–20 nm (Fig. 4A, B). The nanoparticles were generally well-defined and exhibited a relatively narrow size distribution, consistent with previous reports on biosynthesized CeO_2_NPs [20]. Although localized agglomerated clusters were observed, several regions contained well-dispersed nanoparticles, indicating that aggregation was partial rather than extensive. A faint, low-electron-density layer surrounding the nanoparticles was also observed (Fig. 4C). This feature is attributed to an organic corona composed of adsorbed EG and extracellular fungal biomolecules associated with the biosynthesis process. This organic coating may reduce direct particle–particle contact and contribute to the steric stabilization of the nanoparticle surface, which is consistent with the subsequent DLS and zeta potential analyses.

The selected area electron diffraction (SAED) pattern (Fig. 4D) revealed well-defined concentric diffraction rings, confirming the polycrystalline nature of the synthesized nanoparticles. The rings were indexed to the (111), (200), (220), and (311) crystallographic planes of cubic fluorite CeO_2_, demonstrating good crystallinity. The crystalline structure was further confirmed by X-ray diffraction (XRD) analysis (Fig. 4E), which exhibited characteristic diffraction peaks at 2θ = 28.5°, 33.1°, 47.5°, 56.3°, 59.1°, 69.4°, 76.7°, and 79.1°, corresponding to the (111), (200), (220), (311), (222), (400), (331), and (420) crystal planes, respectively. These reflections agree well with the standard cubic fluorite structure of CeO_2_ (JCPDS No. 03-065-5923) and previously published reports [69]. No additional peaks corresponding to secondary phases or impurities were detected, confirming the high phase purity of the biosynthesized EG-CeO₂NPs.

Collectively, the TEM, SAED, and XRD analyses demonstrate the successful biosynthesis of highly crystalline, phase-pure CeO_2_ nanoparticles with nanoscale dimensions and a characteristic cubic fluorite crystal structure.

### DLS and zeta potential analysis

**Fig. 5:**
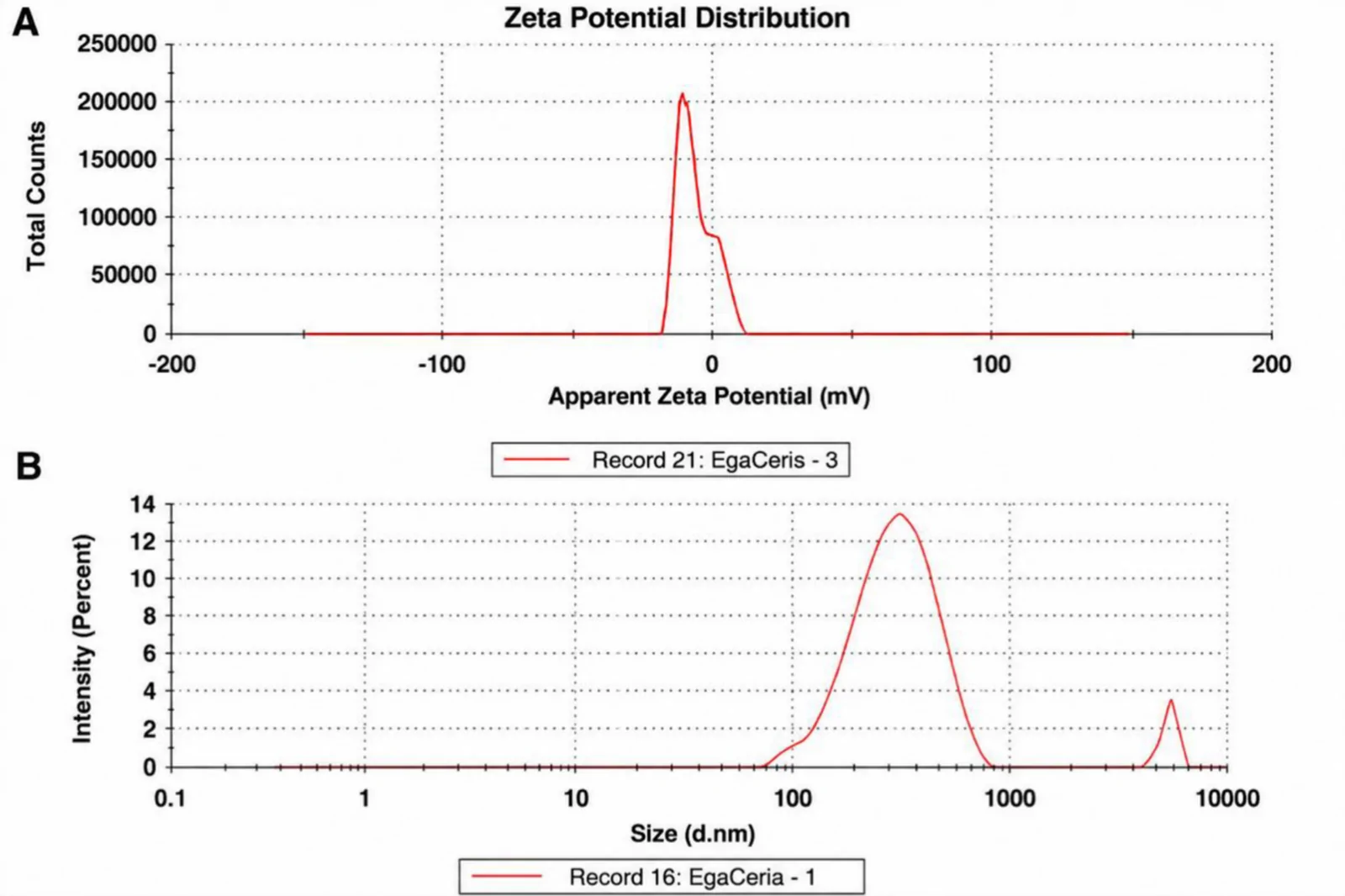
Colloidal characterization of EG-CeO₂NPs. Zeta potential distribution showing a negative surface charge of − 5.43 mV (**A**); DLS analysis illustrating the hydrodynamic size distribution (**B**). Measurements were performed in deionized water at 25 °C with a scattering angle of 173°. The reported PDI values ranged from 0.37 to 0.45, ensuring consistency across triplicate measurements

The hydrodynamic dimensions and surface charge characteristics of the biogenic EG-CeO₂ NPs were evaluated to elucidate their colloidal behavior in aqueous suspension. Dynamic light scattering (DLS) analysis revealed a Z-average hydrodynamic diameter of 571.7–691.3 nm with a polydispersity index (PdI) of 0.37–0.45 (Fig. 5A). It is essential to acknowledge that DLS measures the hydrodynamic diameter, including the hydration shell and organic/biological coating, rather than the primary core size.

A discrepancy was observed between the DLS hydrodynamic diameter and the TEM core size (8–20 nm). This suggests that the nanoparticles partially aggregate in aqueous suspension to form secondary multi-core assemblies. Consequently, the data do not demonstrate excellent colloidal stability; rather, they suggest that aggregation occurs upon hydration, which may influence nanoparticle diffusion dynamics and therapeutic performance [26, 63]. This implies a quasi-heterogeneous state where nanoceria cores associate within an extended hydration layer and biomolecular corona, indicating that further formulation optimization is required.

Zeta potential profiling showed a weakly negative surface charge of − 5.43 mV (Fig. 5B). Since absolute values below ± 30 mV reflect low electrostatic repulsion, the nanoparticles are considered weakly electrostatically stabilized. Although they exhibit kinetic dispersibility, their stabilization is hypothesized to rely on steric hindrance mediated by the EG and fungal biomolecular capping layer [26, 70]; however, in the absence of direct surface-force quantification, this remains a proposed hypothesis rather than an experimentally confirmed mechanism.

While these hydrodynamic dimensions may limit deep tissue transmigration, this structural organization could facilitate a localized functional role. In cystic fibrosis (CF) respiratory mucus, the near-neutral surface potential (− 5.43 mV) and hydrophilic EG corona may minimize non-specific mucoadhesion and passivation against polyanionic mucin entrapment [71–73]. We hypothesize that these kinetically stable aggregates remain sequestered within superficial mucus and biofilm boundaries, functioning as localized therapeutic reservoirs—a hypothesis that warrants future experimental investigation.

### EG-CeO₂NPs suppress the proliferation of *P. aeruginosa*

Previous studies have highlighted the broad-spectrum antibacterial efficacy of CeO_2_ NPs against diverse Gram-positive and Gram-negative pathogens [74, 75]. In the present study, the susceptibility of XDR *P. aeruginosa* isolates to various concentrations of EG-CeO₂NPs (2.5, 5, and 10 mg/mL) was evaluated. The synthesized nanoparticles exhibited robust antibacterial activity, with inhibition zones ranging from 29 to 32 mm across all tested isolates (Table 1). Notably, the PA-5 isolate demonstrated the highest sensitivity, yielding a maximum inhibition zone of 32 mm at the 10 mg/mL concentration. Furthermore, a clear dose-dependent response was observed, as the diameter of the inhibition zones increased proportionally with nanoparticle concentration [76]. In contrast, the EG control did not exhibit detectable antibacterial activity under the tested conditions.

**Table 1:** Antibacterial activity of EG-CeO_2_NPs against XDR *P. aeruginosa*

Bacteria	Inhibition zone (mm)			MIC mg/mL	MBC mg/mL
	Concentration (mg/mL)				
	2.5	5	10		
PA-1	13^a^ ± 0.16	20^b^ ± 0.37	29^c^ ± 0.22	1.25	2.50
PA-2	14 ^a^ ± 0.28	20 ^b^ ± 0.37	30 ^c^ ± 0.65	0.60	1.25
PA-3	15 ^a^ ± 0.08	21 ^b^ ± 0.42	30^c^ ± 0.57	1.25	2.50
PA-4	15 ^a^ ± 0. 24	22 ^b^ ± 0.57	29 ^c^ ± 0.41	1.25	2.50
PA-5	17 ^a^ ± 0.57	25 ^b^ ± 0.08	32 ^c^ ± 0.64	0.60	1.25
PA-6	15 ^a^ ± 0.00	22 ^b^ ± 0.29	31 ^c^ ± 0.36	0.60	1.25
PA-ATCC 27853	18 ^a^ ± 0.14	27 ^b^ ± 0.43	33 ^c^ ± 0.00	1.25	2.50

Values are presented as mean ± SD (*n* = 3). Different superscript letters within the same row (or across concentrations for each bacterial strain) indicate significant differences according to one-way ANOVA followed by Dunnett’s multiple comparisons test (*P* < 0.05). Ethylene glycol (EG) alone (10 mg/mL) exhibited no zone of inhibition across all tested strains

*MIC* minimum inhibitory concentration, *MBC* minimum bactericidal concentration, *XDR* extensively drug-resistant

The superior antibacterial efficacy of EG-CeO₂NPs is primarily attributed to their reduced particle size, which facilitates rapid adsorption onto microbial surfaces compared to bulk counterparts. Historically, the antimicrobial actions of metal oxide nanoparticles, specifically CeO_2_, have been linked to multiple distinct pathways [77–79]. As elucidated by Zhang et al. [80], these mechanisms often involve direct physical contact with the bacterial envelope or the induction of secondary cytotoxic byproducts. A predominant factor in this bactericidal process is the production of reactive oxygen species (ROS), which triggers extensive oxidative damage and subsequent cellular demise [74, 78]. Furthermore, the adsorption of EG-CeO₂NPs onto the bacterial membrane may disrupt vital physiological functions, including mesosomal integrity, cellular respiration, DNA replication, and binary fission, ultimately culminating in cell death [81].

The MIC of the synthesized EG-CeO₂NPs was determined across the tested XDR *P. aeruginosa* strains. The nanoparticles demonstrated robust inhibitory efficacy, with MIC values ranging from 0.625 to 1.25 mg/mL. In all cases, the minimum bactericidal concentration (MBC) was twofold the corresponding MIC (Table 1), indicating a clear bactericidal mode of action. Notably, the antibacterial potency observed in this study surpasses several previously reported values for CeO_2_NPs. For instance, prior literature has documented significantly higher MICs, such as 2.15 to 10 mg/mL against *E. coli* and *S. aureus* [82], and up to 50–100 mg/mL in other investigations [83]. This enhanced performance is attributed to the superior stability and surface properties provided by the EG capping.

**Fig. 6:**
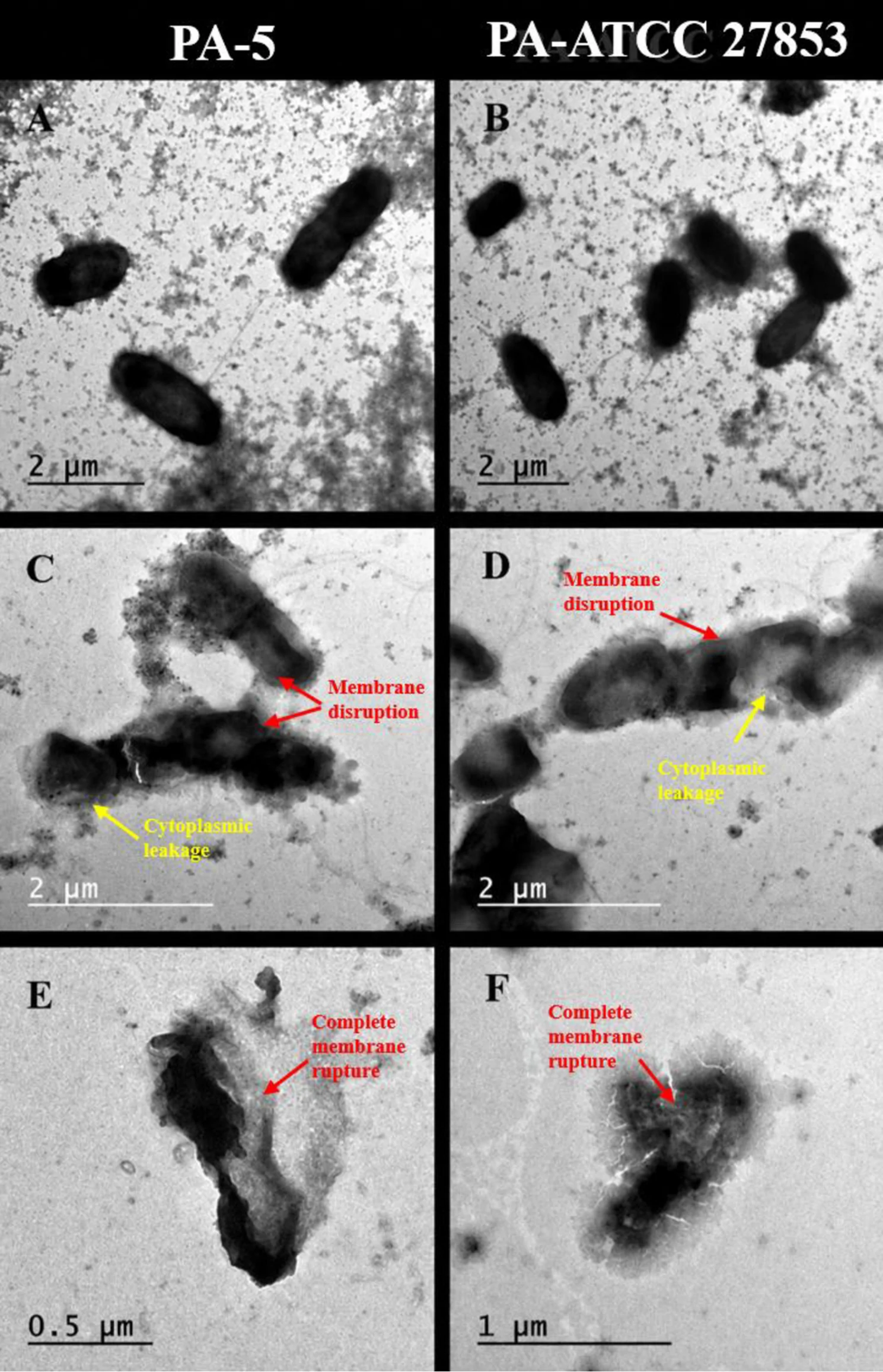
TEM analysis of *P. aeruginosa* (PA-5 and PA-ATCC 27853). **A**, **B** Untreated cells with intact walls. **C**, **D** Cells treated at ½ MIC showing membrane disruption (red arrows) and cytoplasmic leakage (yellow arrows). **E**, **F** Cells treated at MIC showing complete rupture and cytoplasmic release (red arrows). Images represent multiple independent grid analyses of the nanoparticle preparation. Magnification: 5000×–30,000×

The impact of EG-CeO₂NPs on bacterial ultrastructure was further elucidated through TEM analysis. Upon exposure, significant morphological alterations were observed, including the disruption of cell membrane integrity, extensive cellular disintegration, and the subsequent leakage of cytoplasmic constituents, ultimately leading to irreversible cell death (Fig. 6). Collectively, these microscopic observations corroborate the potent antibacterial efficacy of EG-CeO₂NPs against XDR *P. aeruginosa*.

**Fig. 7:**
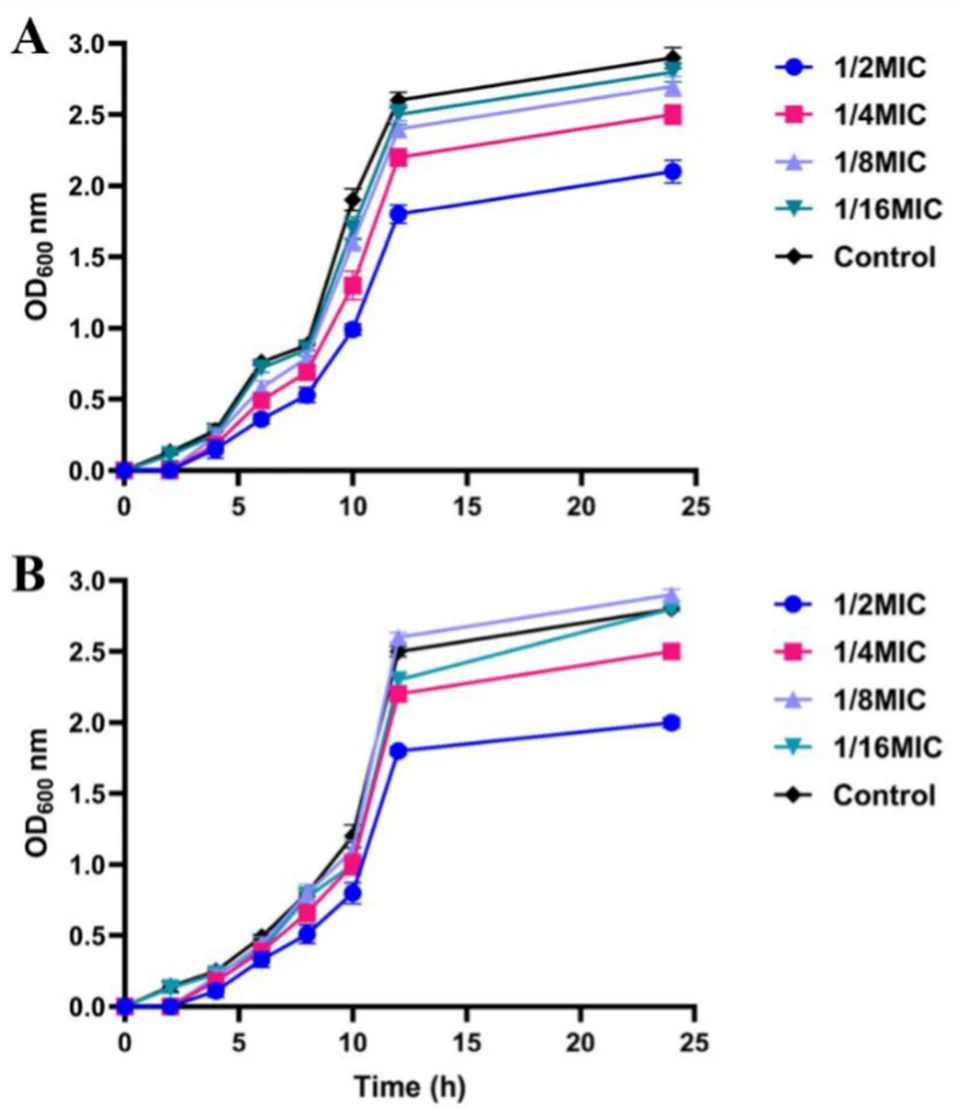
Growth kinetics of *P. aeruginosa* PA-5 (**A**) and ATCC 27853 (**B**) under sub-MIC EG-CeO₂NPs. OD₆₀₀ monitored over 24 h (mean ± SD, *n* = 3). Significance determined by two-way ANOVA with Dunnett’s test (*P* < 0.0001)

To evaluate the non-lethal impact of biosynthesized EG-CeO₂NPs, the growth kinetics of *P. aeruginosa* PA-5 and ATCC 27853 were monitored under sub-MIC treatments (1/2, 1/4, 1/8, and 1/16 × MIC) over 24 h. As illustrated (Fig. 7A, B, and Table S9, 10), the nanoparticles induced a concentration-dependent delay in bacterial growth. Statistical analysis using two-way ANOVA confirmed that incubation time, treatment concentration, and their interaction had highly significant effects on bacterial growth (*P* < 0.0001). Incubation time was identified as the primary source of variation, accounting for approximately 95.78% and 96.79% of the total variation for both strains, respectively. During the early lag phase (2–4 h), significant growth reduction was observed; however, by the 4th hour, this effect remained statistically significant primarily for the 1/2 MIC treatment (*P* < 0.01 to *P* < 0.001). Upon entering the exponential growth phase (6–10 h), the divergence from the control group became more pronounced. Notably, at the 10 h mark, all tested sub-MIC concentrations, including the lowest (1/16 × MIC), exhibited highly significant growth retardation (*P* < 0.0001) in both strains. During the stationary phase (12–24 h), growth suppression remained highly significant for the 1/2 and 1/4 × MIC treatments (*P* < 0.0001). In contrast, the 1/16 × MIC-treated cultures gradually recovered, merging back toward control levels with no statistically significant differences (*P* > 0.05) by the end of the 24 h incubation period. These findings demonstrate that while 1/2 × MIC significantly delays growth kinetics, it does not compromise bacterial viability, confirming its suitability for subsequent non-lethal anti-virulence investigations.

### EG-CeO₂NPs induce oxidative stress in *P. aeruginosa*

**Fig. 8:**
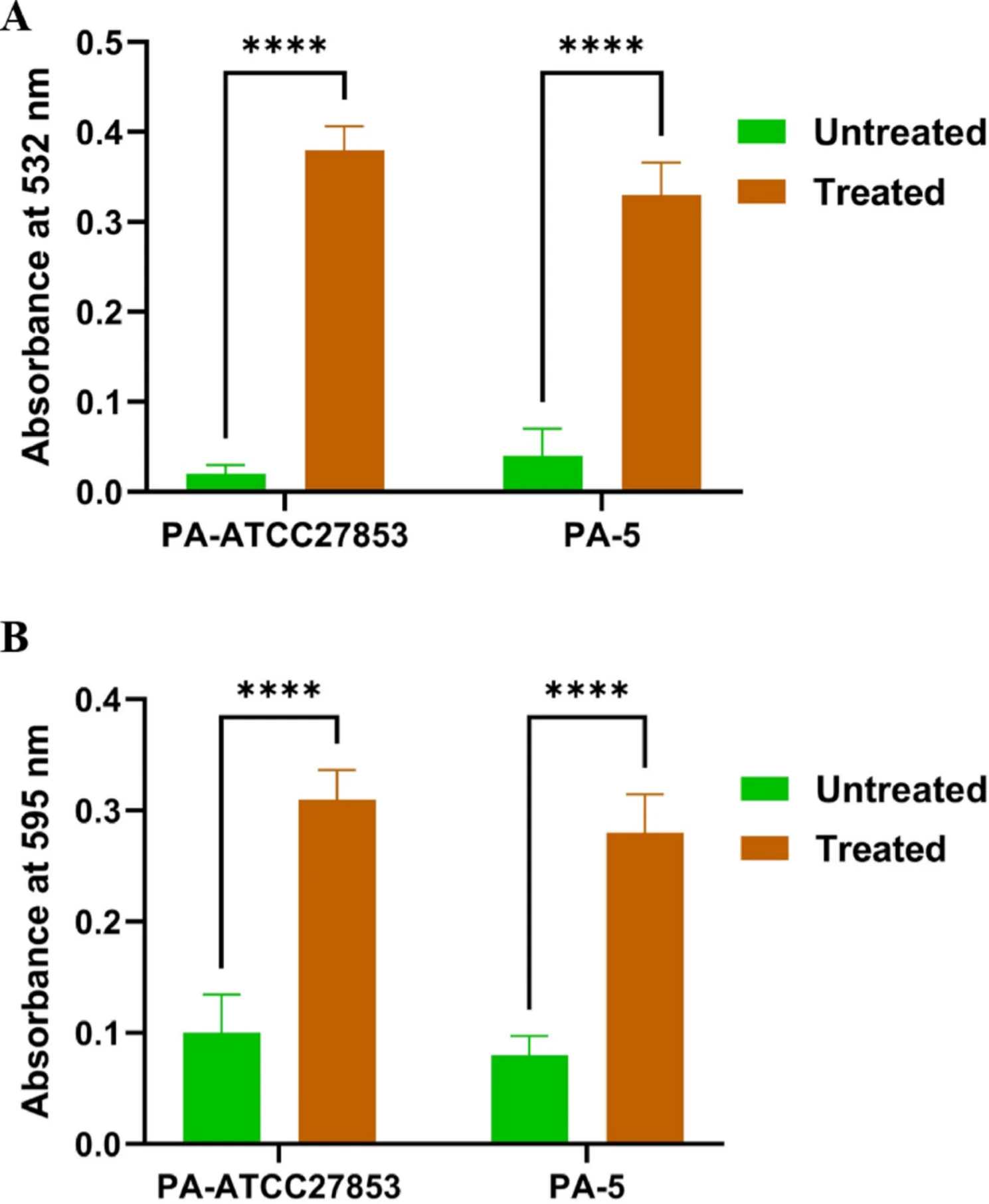
Oxidative stress and membrane damage induced by EG-CeO₂NPs in *P. aeruginosa*. **A** Lipid peroxidation via MDA levels (532 nm). **B** Protein leakage via absorbance (595 nm). Data are mean ± SD (*n* = 3). Significance determined by two-way ANOVA with Dunnett’s test (**** P < 0.0001)

Lipid peroxidation serves as a robust biochemical indicator of ROS accumulation under nanoparticle-induced stress [84, 85]. Herein, the involvement of an oxidative stress-related mechanism in the bactericidal action of EG-CeO₂NPs was indicated by quantifying malondialdehyde (MDA) production. As a primary byproduct of polyunsaturated fatty acid degradation, MDA reflects the extent of putative ROS-mediated damage to the bacterial cytoplasmic membrane. Our results demonstrated a significant elevation in MDA levels in both *P. aeruginosa* ATCC 27853 and PA-5 strains upon treatment with 1/2 ×MIC of EG-CeO₂NPs (Fig. 8A). This oxidative surge, acting through proposed mechanisms supported by previous literature [86, 87], likely overwhelms the bacterial antioxidant defense system, triggering a cascade of lipid peroxide radical formation and subsequent membrane rupture. These findings imply that the bactericidal activity of EG-CeO₂NPs is driven by a dual-action mechanism involving direct physical membrane compromise and inferred intracellular oxidative damage, consistent with previously reported effects of silver nanoparticles on *E. coli* and *P. aeruginosa* [88].

### Intracellular protein leakage after treatment with EG-CeO₂NPs

Disruption of membrane integrity exerts a profound impact on membrane potential and associated bioenergetic conversion systems [89]. This phenomenon was validated through the quantification of intracellular protein leakage. As illustrated in Fig. 8B, treatment with EG-CeO₂NPs at 1/2 ×MIC led to a significant surge in protein efflux for both *P. aeruginosa* strains compared to control groups. Such pronounced leakage serves as a definitive marker for the deterioration of bacterial envelope permeability and the loss of structural integrity. These observations align with findings by Du et al. [90], who reported that manganese dioxide nanosheets facilitate bacterial eradication via ROS-mediated membrane perforation and subsequent discharge of cytoplasmic constituents. Consequently, we postulate that EG-CeO₂NPs initially compromise the bacterial cell wall, increasing permeability and facilitating the internal accumulation of nanomaterials, which is proposed to further amplify reactive oxygen species (ROS) generation, putatively culminating in cell death. Statistical analysis confirmed that both lipid peroxidation and protein leakage were significantly increased in treated bacteria compared with untreated controls (*P* < 0.0001), indicating severe oxidative membrane damage.

Based on the collective findings of the present study, the observed antibacterial and antivirulence activities of the biogenic EG-CeO₂NPs can be mechanistically linked to their distinct physicochemical profile. The small particle size (8–20 nm) observed via TEM provides a high surface-area-to-volume ratio, maximizing the contact interface with the bacterial cell wall and facilitating rapid adsorption. Furthermore, the surface chemistry characterized by a mixed-valence state (Ce³⁺/Ce⁴⁺) and oxygen vacancies acts as a redox-active catalyst. These surface defects are proposed to continuously generate ROS, which likely triggers the observed lipid peroxidation and irreversible membrane disruption, as supported by previous literature on nanoceria redox mechanisms. The polycrystalline cubic fluorite structure confirmed by XRD ensures a stable lattice that supports these reactive sites without structural degradation. Finally, surface functionalization with the EG-organic corona plays a crucial role; beyond providing steric stability, it modulates interaction with the bacterial quorum-sensing machinery. The hydrophilic, near-neutral surface chemistry likely facilitates the proximity of nanoparticles to signaling molecules and membrane-bound receptors, enhancing the downregulation of QS regulatory genes (*lasR/I* and *rhlR/I*) and associated virulence factors.

### EG-CeO₂NPs inhibits the process of biofilm development

**Fig. 9:**
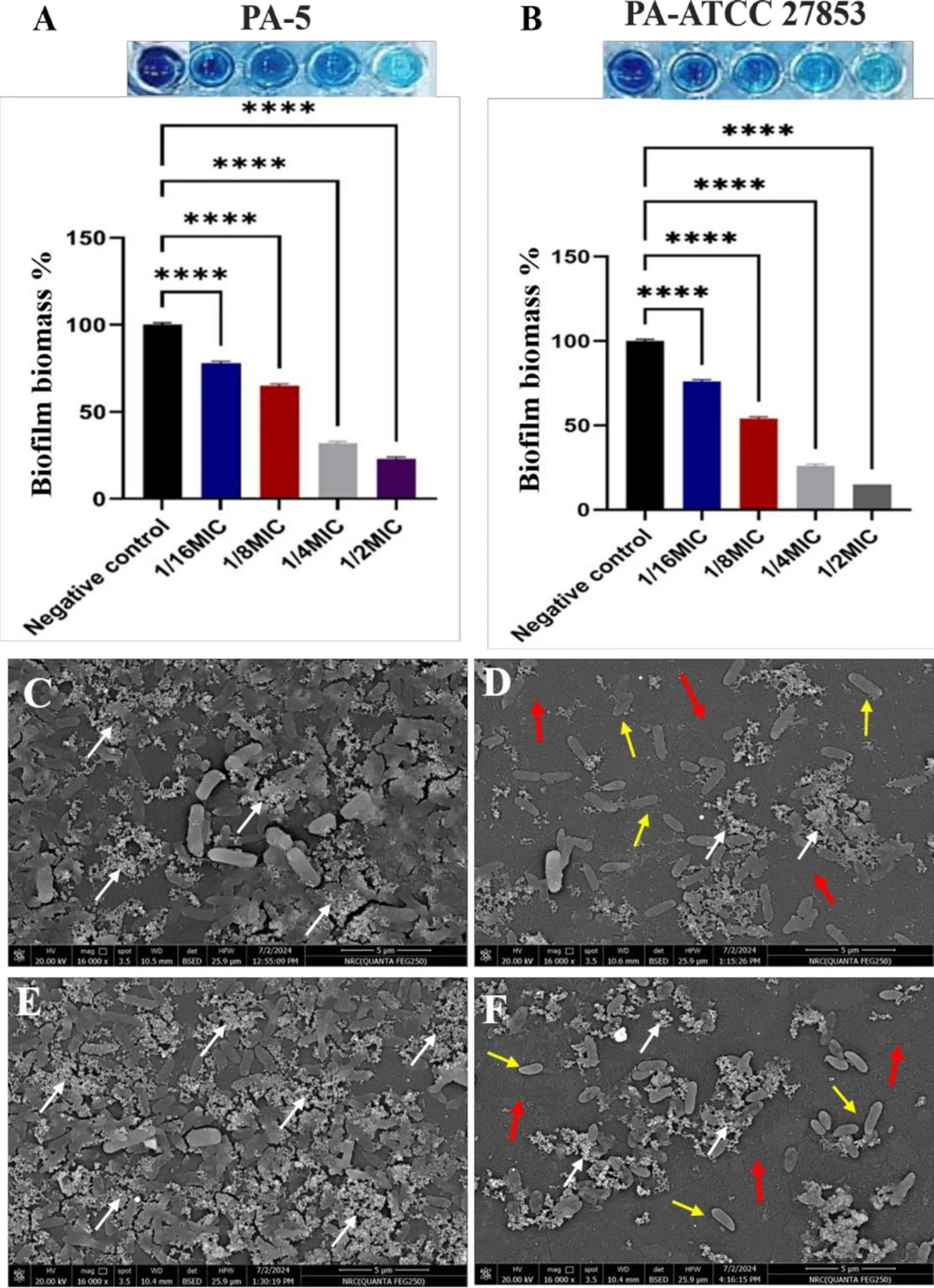
Antibiofilm efficacy of EG-CeO₂NPs against *P. aeruginosa* PA-5 and PA-ATCC 27853. **A**, **B** Biofilm inhibition. Data are mean ± SD (*n* = 3). Significance determined by one-way ANOVA with Dunnett’s test (****P < 0.0001). **C**–**F** FE-SEM micrographs (16,000×): **C**, **E** Untreated control showing dense communities and intact EPS (white arrows); **D**, **F** treated biofilm (½ MIC) showing cell depletion (yellow arrows) and structural disintegration (red arrows)

Biofilms represent a sophisticated survival strategy for pathogenic bacteria, affording heightened resilience against environmental stressors and facilitating persistent host colonization [91]. The orchestration of biofilm architecture is intricately linked to the Quorum Sensing (QS) system, and it is estimated that approximately 80% of human microbial infections are associated with biofilm-protected communities [92]. Consequently, identifying novel bioactive agents capable of disrupting these structures is a clinical priority. In this study, the antibiofilm efficacy of EG-CeO₂NPs was evaluated at sub-MICs against *P. aeruginosa* PA-5 and ATCC 27853 (Fig. 9A, B). Our findings characterize EG-CeO₂NPs as a potent antibiofilm agent, significantly attenuating the initiation of new biofilm matrices (*P* < 0.001). Importantly, at these sub-MIC levels, bacterial proliferation remained unaffected, indicating that the observed reduction in biofilm formation is a specific anti-virulence response rather than a consequence of direct bactericidal activity.

The quantitative biofilm inhibition data were visually corroborated through FE-SEM analysis. Control samples for both *P. aeruginosa* PA-5 and ATCC 27853 strains exhibited the formation of extensive, dense cellular aggregates characterized by thick clusters encased within an exopolymeric substance (EPS) matrix (Fig. 9C, D). Conversely, treatment with EG-CeO₂NPs at ½ × MIC led to a profound reduction in both cellular density and the degree of aggregation. A significant attenuation of the EPS matrix was also evident, as depicted in (Fig. 9E, F) where cells appeared scattered and lacked the protective encasement typical of mature biofilms. These ultrastructural observations provide compelling evidence for the capacity of EG-CeO₂NPs to disrupt biofilm architecture. Our findings are consistent with previous reports demonstrating the antibiofilm efficacy of various metallic nanoparticles, such as AgNPs, ZnONPs, and Ti-Ce nanocomposites, against *P. aeruginosa* [93–96]. Nevertheless, further mechanistic investigations are warranted to fully elucidate the specific molecular pathways by which these nanoparticles exert their antibiofilm action.

### EG-CeO₂NPs eradicate the established biofilm of *P. aeruginosa*

**Fig. 10:**
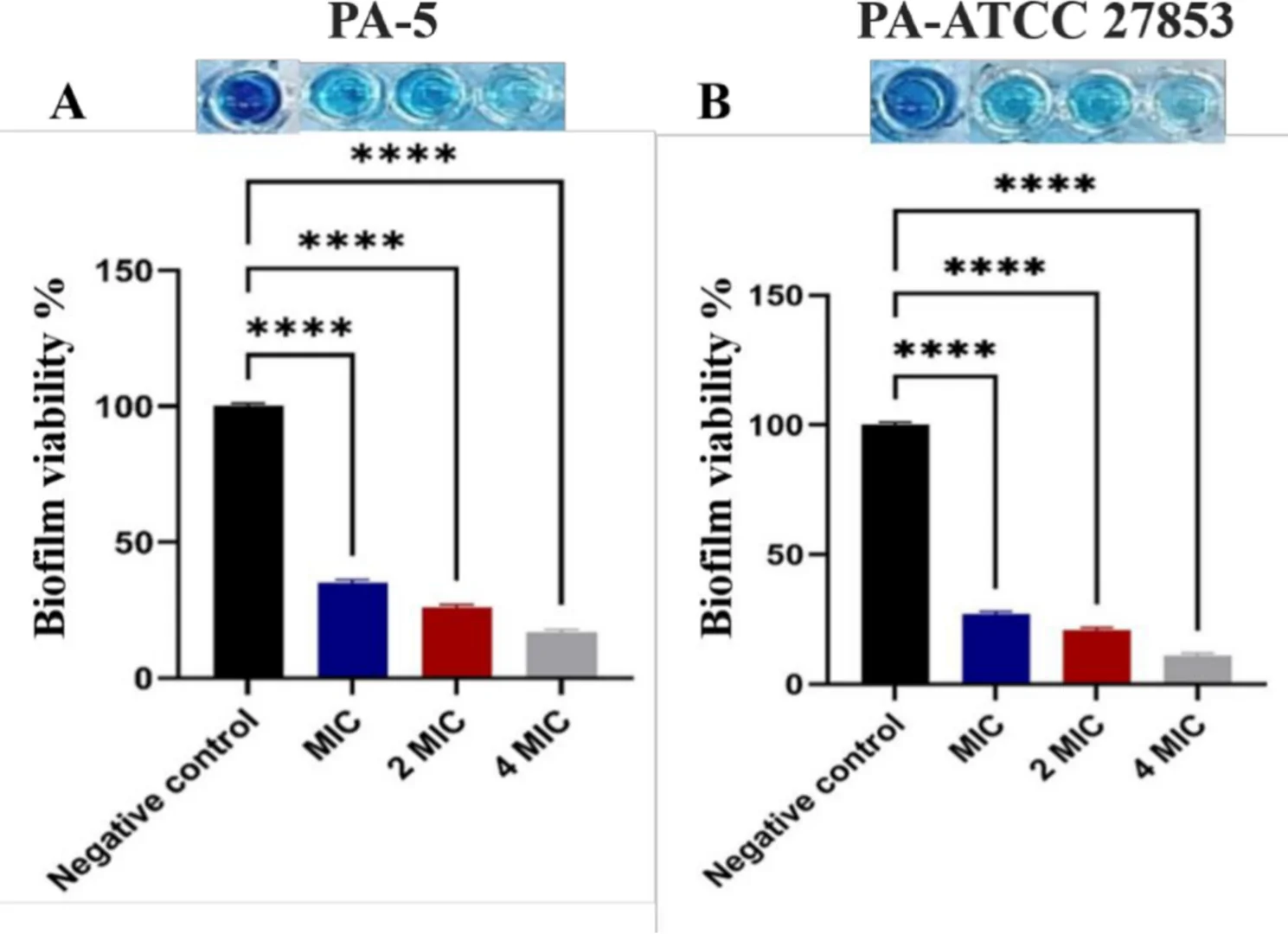
Eradication of mature *P. aeruginosa* biofilms by EG-CeO₂NPs. **A**, **B** Dose-dependent reduction in biofilm biomass for PA-5 and PA-ATCC 27853. Data are mean ± SD (*n* = 3). Significance determined by one-way ANOVA followed by Dunnett’s test (****P < 0.0001)

Conventional antimicrobial therapies predominantly target planktonic populations, often failing to impede or penetrate the structural defenses of established biofilms [97, 98]. In this study, we evaluated the capacity of EG-CeO₂NPs to eradicate mature, pre-formed biofilms of *P. aeruginosa*. While sub-MICs were effective in preventing biofilm initiation, supra-inhibitory concentrations (MIC, 2× MIC, and 4× MIC) demonstrated a potent, dose-dependent eradication profile (Fig. 10). Quantitative analysis via the microtiter plate (MTP) assay revealed that treatment at MIC, 2× MIC, and 4× MIC significantly reduced the biofilm biomass of the PA-5 isolate by 65%, 74%, and 83%, respectively. Similarly, the reference strain showed even higher susceptibility, with reductions of 73%, 79%, and 89% (Fig. 10A, B). Furthermore, the robust anti-biofilm and antibacterial dynamics observed in our study corroborate recent reports on biopolymer-mediated nanoceria architectures. For instance, CeO_2_ NPs green-synthesized utilizing alginate demonstrated significant growth inhibition against pathogenic strains, alongside a distinct capacity to disrupt established biofilm structures [98].

Notably, our results surpass the efficacy reported by Altaf et al. [95], where a titanium-cerium nanocomposite achieved up to 73% eradication. This outstanding performance further validates that our surface-stabilized nanoceria successfully compromises biofilm integrity, maximizing the therapeutic efficiency of biogenic cerium nanostructures against recalcitrant bacterial communities compared to traditional metal-composite alternatives. The superior performance of EG-CeO₂NPs suggests a multi-mechanistic disruption involving interactions with the extracellular polymeric substance (EPS) matrix, eDNA, and Quorum Sensing (QS) signaling pathways, which destabilizes the biofilm architecture and compromises the viability of sequestered cells [99–103].

### EG-CeO₂NPs modulate virulence-associated gene expression in *P. aeruginosa*

The impact of EG-CeO₂NPs on the transcriptional profile of quorum sensing (QS), biofilm-associated, and virulence-related genes in the PA-5 isolate was evaluated using qRT-PCR under sub-inhibitory concentrations. *P. aeruginosa* possesses three interconnected QS systems (*Las*,* Rhl*, and *Pqs*), which coordinately regulate the expression of multiple virulence determinants and play a pivotal role in infection establishment [104]. Targeting these regulatory networks is considered an effective strategy to attenuate bacterial pathogenicity without exerting strong selective pressure for resistance [105].

**Fig. 11:**
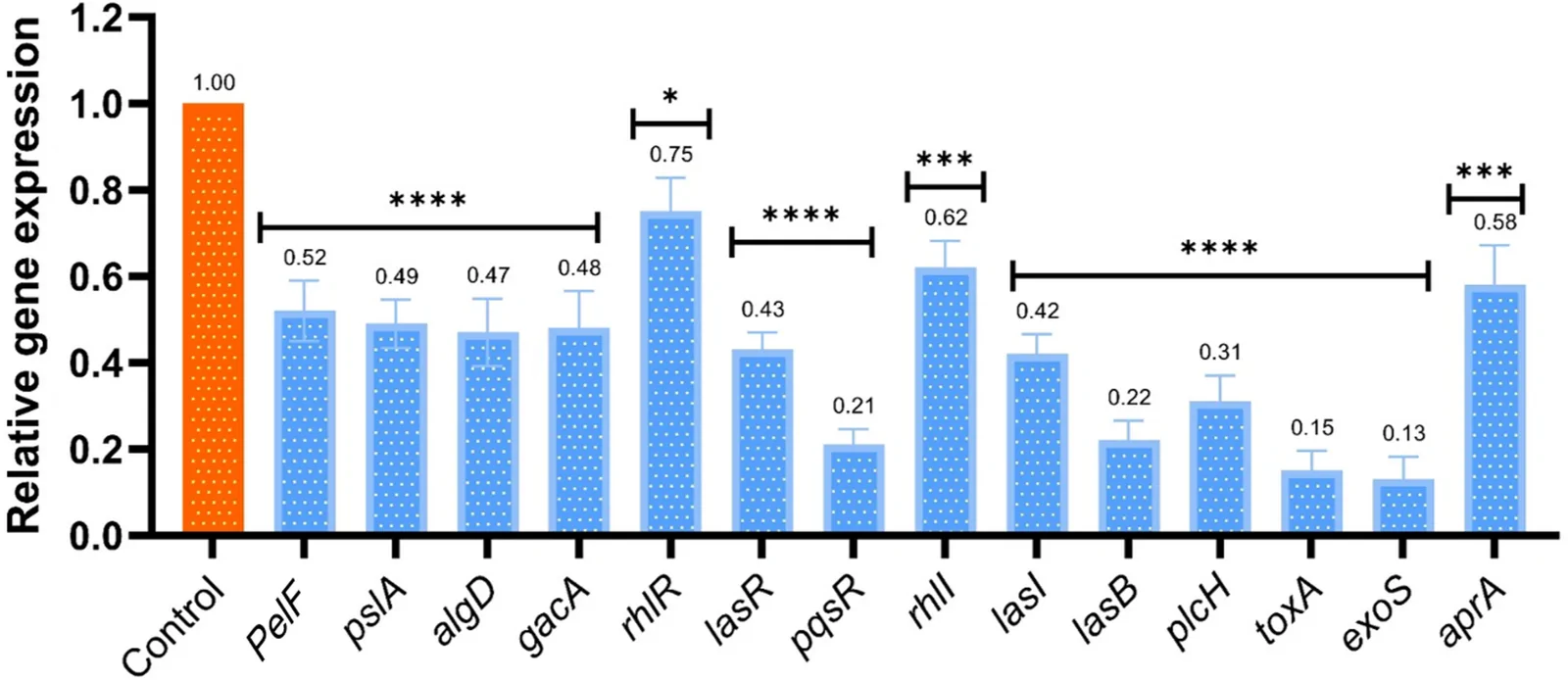
RT-qPCR analysis of virulence genes in *P. aeruginosa* PA-5 treated with EG-CeO₂NPs (½ MIC). Data represent the relative expression (fold change) of genes as mean ± SD of three independent experiments. Statistical significance determined by one-way ANOVA followed by Dunnett’s test (****P < 0.0001)

As illustrated in (Table S11 & Fig. 11), treatment with EG-CeO₂NPs resulted in a marked downregulation of key QS regulatory genes, including *lasR/I*,* rhlR/I*, and *pqsR*, with relative gene expression decreasing to values ranging from 0.21 to 0.75 (corresponding to a 25% to 79% reduction) compared to untreated controls. These findings suggest that EG-CeO₂NPs interfere with QS signaling pathways, thereby impairing the coordinated expression of virulence factors. Given the strong association between QS signaling and biofilm development, the expression of major biofilm-related genes was also investigated [106]. These genes *pelF*,* pslA*,* algD*, and *gacA*, which are involved in the synthesis and regulation of essential exopolysaccharides, were significantly downregulated following nanoparticle treatment (*p* < 0.0001), This indicates a substantial disruption of biofilm matrix formation and structural integrity.

In addition to QS and biofilm-related genes, several critical virulence factors were also affected. The expression of *exoS* and *toxA*, encoding exoenzyme S and exotoxin A, respectively, was significantly reduced to relative expression levels of 0.13 and 0.15 (87% and 85% reduction) upon treatment with EG-CeO₂NPs at ½ MIC. These toxins are known to contribute to host cell damage, immune evasion, and disease progression. Similarly, the expression of *plcH*, which encodes a hemolytic phospholipase involved in the degradation of host membrane components [107–110], was significantly decreased. Moreover, the protease-encoding genes *lasB* and *aprA*, which facilitate tissue invasion through degradation of host proteins [111, 112], were also downregulated to relative expression levels of 0.22 and 0.58 (78% and 42% reduction, respectively; *p* < 0.0001). The molecular findings were consistent with the phenotypic observations obtained from the crystal violet biofilm inhibition assay and SEM imaging. The downregulation of key biofilm-associated genes (*pel*F, *psl*A, *alg*D, and *gac*A), together with the suppression of quorum sensing regulators (*las*R, *las*I, *rhl*R, *rhl*I, and *pqs*R), correlated well with the marked reduction in biofilm biomass and disruption of biofilm observed in Fig. 9. These results suggest that EG-CeO₂NPs inhibit biofilm development through coordinated suppression of both EPS biosynthesis and quorum sensing regulatory networks.

These findings are consistent with previous study demonstrating the ability of metal oxide nanoparticles to suppress QS-regulated virulence pathways in *P. aeruginosa* [113]. Collectively, the observed transcriptional suppression of QS, biofilm, and virulence genes indicates that EG-CeO₂NPs exert a multi-targeted anti-virulence effect, which is attributable to their capacity to induce oxidative stress, disrupt membrane integrity, and interfere with intracellular regulatory systems, ultimately leading to reduced pathogenicity.

### Toxicity and translational limitations

Although EG-CeO₂NPs demonstrated promising antimicrobial and antibiofilm activities, the present study has several limitations. The safety assessment was primarily based on in silico ADMET predictions, which provide only preliminary evidence and cannot substitute for experimental toxicological evaluation. Therefore, comprehensive in vitro cytotoxicity studies using relevant pulmonary cell lines (e.g., A549 human alveolar epithelial cells), followed by in vivo investigations, are mandatory before considering clinical translation.

Regarding the relatively large hydrodynamic diameter observed by DLS (600–700 nm), these results confirm that the nanoparticles exist as aggregated nanoparticle assemblies in aqueous media rather than as individual primary units. While this indicates partial aggregation, the present data do not establish whether such aggregation is advantageous or detrimental for respiratory applications. Previous studies have suggested that surface-functionalized nanoparticle aggregates may exhibit prolonged interactions with mucus and biofilm matrices; however, these properties were not evaluated in the current study and require dedicated investigation. Accordingly, the present work should be interpreted as a predictive proof-of-concept demonstrating the antimicrobial potential of EG-CeO₂NPs, rather than a definitive confirmation of their clinical safety, colloidal stability under physiological conditions, or monodispersity.

## Conclusion

This study establishes a novel biotechnological framework by utilizing the endophytic yeast *C. lusitaniae* as a highly efficient microbial cell factory for the biogenic synthesis of CeO₂NPs, followed by surface capping with EG. The integration of fungal-mediated bioprocessing with surface engineering yielded a prospective nanoplatform characterized by distinct physicochemical properties and aggregated colloidal behavior in aqueous suspension. Biological validation demonstrated the potent, multi-targeted efficacy of these nanoparticles against XDR *P. aeruginosa*, achieving significant disruption of membrane integrity and biofilm architecture. Molecular analyses confirmed that these microbial-derived nanoparticles effectively modulate bacterial pathogenicity by downregulating critical quorum-sensing and virulence gene networks. These findings underscore the potential of endophyte-based biological factories as a sustainable and scalable strategy for producing advanced nanomaterials, offering a promising antimicrobial approach for combating recalcitrant respiratory infections. While *C. lusitaniae* effectively synthesizes functionalized CeO₂NPs, clinical translation remains theoretical. Overcoming physiological barriers, such as sputum viscosity, and evaluating pulmonary biocompatibility and potential toxicity are critical. Therefore, rigorous in vitro (e.g., A549 cells) and in vivo preclinical evaluations are mandatory to establish a definitive safety profile before integrating these biogenic nanostructures into nanomedicine for the treatment of recalcitrant respiratory infections. In addition, further studies are required to evaluate colloidal behavior under physiologically relevant conditions and to determine whether the nanoparticle aggregates exhibit favorable mucus and biofilm interactions in respiratory environments.

## Supplementary Information

Below is the link to the electronic supplementary material.

Supplementary Material 1.

## Data Availability

All data generated or analyzed during this study are included in this published article.

## References

[1] Maillard J-Y, Bloomfield SF, Courvalin P, Essack SY, Gandra S, Gerba CP, et al. Reducing antibiotic prescribing and addressing the global problem of antibiotic resistance by targeted hygiene in the home and everyday life settings: a position paper. Am J Infect Control. 2020;48:1090–9. 10.1016/j.ajic.2020.04.011. 32311380 10.1016/j.ajic.2020.04.011 PMC7165117

[2] O’Neill J. Review on antimicrobial resistance: tackling drug-resistant infections globally: final report and recommendations. 2016.

[3] Gellatly SL, Hancock RE. *Pseudomonas aeruginosa*: new insights into pathogenesis and host defenses. Pathog Dis. 2013;67:159–73. 10.1111/2049-632X.12033. 23620179 10.1111/2049-632X.12033

[4] Koch C. Early infection and progression of cystic fibrosis lung disease. Pediatr Pulmonol. 2002;34:232–6. 10.1002/ppul.10135. 12203855 10.1002/ppul.10135

[5] Engel JN. Molecular pathogenesis of acute *Pseudomonas aeruginosa* infections. Sev Infect Caused *Pseudomonas aeruginosa*. 2003. p. 201–29. 10.1007/978-1-4615-0433-7_13

[6] Osmon S, Ward S, Fraser VJ, Kollef MH. Hospital mortality for patients with bacteremia due to *Staphylococcus aureus* or *Pseudomonas aeruginosa*. Chest. 2004;125:607–16. 10.1378/chest.125.2.607. 14769745 10.1378/chest.125.2.607

[7] Lynch JP III, Zhanel GG, Clark NM. Emergence of antimicrobial resistance among *Pseudomonas aeruginosa*: implications for therapy. 2017. pp. 326–45. 10.1055/s-0037-1602583 10.1055/s-0037-1602583 28578556

[8] Potron A, Poirel L, Nordmann P. Emerging broad-spectrum resistance in *Pseudomonas aeruginosa* and *Acinetobacter baumannii*: mechanisms and epidemiology. Int J Antimicrob Agents. 2015;45:568–85. 10.1016/j.ijantimicag.2015.03.001. 25857949 10.1016/j.ijantimicag.2015.03.001

[9] McCarthy K. *Pseudomonas aeruginosa*: evolution of antimicrobial resistance and implications for therapy. 2015. pp. 044–55. 10.1055/s-0034-1396907 10.1055/s-0034-1396907 25643270

[10] Livermore DM. Has the era of untreatable infections arrived? J Antimicrob Chemother. 2009;64:i29–36. 10.1093/jac/dkp255. 19675016 10.1093/jac/dkp255

[11] Peña C, Suarez C, Gozalo M, Murillas J, Almirante B, Pomar V, et al. Prospective multicenter study of the impact of carbapenem resistance on mortality in *Pseudomonas aeruginosa* bloodstream infections. Antimicrob Agents Chemother. 2012;56:1265–72. 10.1128/AAC.05991-11. 22155832 10.1128/AAC.05991-11 PMC3294876

[12] Oliver A, Mena A, Maciá MD Evolution of *Pseudomonas aeruginosa* pathogenicity: from acute to chronic infections. Evol Biol Bact Fungal Pathog. 2007. 10.1128/9781555815639.ch36.

[13] Sousa AM, Pereira MO. *Pseudomonas aeruginosa* diversification during infection development in cystic fibrosis lungs—a review. Pathogens. 2014;3:680–703. 10.3390/pathogens3030680. 25438018 10.3390/pathogens3030680 PMC4243435

[14] Winstanley C, O’Brien S, Brockhurst MA. *Pseudomonas aeruginosa* evolutionary adaptation and diversification in cystic fibrosis chronic lung infections. Trends Microbiol. 2016;24:327–37. 10.1016/j.tim.2016.01.008. 26946977 10.1016/j.tim.2016.01.008 PMC4854172

[15] Moreau-Marquis S, Stanton BA, O’Toole GA. *Pseudomonas aeruginosa* biofilm formation in the cystic fibrosis airway. Pulm Pharmacol Ther. 2008;21:595–9. 10.1016/j.pupt.2007.12.001. 18234534 10.1016/j.pupt.2007.12.001 PMC2542406

[16] Høiby N, Bjarnsholt T, Givskov M, Molin S, Ciofu O. Antibiotic resistance of bacterial biofilms. Int J Antimicrob Agents. 2010;35:322–32. 10.1016/j.ijantimicag.2009.12.011. 20149602 10.1016/j.ijantimicag.2009.12.011

[17] Thi MTT, Wibowo D, Rehm BH. *Pseudomonas aeruginosa* biofilms. Int J Mol Sci. 2020;21:8671. 10.3390/ijms21228671. 33212950 10.3390/ijms21228671 PMC7698413

[18] Hassett DJ, Korfhagen TR, Irvin RT, Schurr MJ, Sauer K, Lau GW, et al. *Pseudomonas aeruginosa* biofilm infections in cystic fibrosis: insights into pathogenic processes and treatment strategies. Expert Opin Ther Targets. 2010;14:117–30. 10.1517/14728220903454988. 20055712 10.1517/14728220903454988

[19] Han C, Romero N, Fischer S, Dookran J, Berger A, Doiron AL. Recent developments in the use of nanoparticles for treatment of biofilms. Nanotechnol Rev. 2017;6:383–404. 10.1515/ntrev-2016-0054.

[20] Thill A, Zeyons O, Spalla O, Chauvat F, Rose J, Auffan M, et al. Cytotoxicity of CeO2 nanoparticles for *Escherichia coli.* Physico-chemical insight of the cytotoxicity mechanism. Environ Sci Technol. 2006;40:6151–6. 10.1021/es060999b. 17051814 10.1021/es060999b

[21] Das S, Dowding JM, Klump KE, McGinnis JF, Self W, Seal S. Cerium oxide nanoparticles: applications and prospects in nanomedicine. Nanomed. 2013;8:1483–508. 10.2217/nnm.13.133. 10.2217/nnm.13.133 23987111

[22] Shariff MZ, Mallegowda DG, Ningegowda R, Kameshwar VH. Endophyte-mediated biosynthesis of biogenic nanoparticles: mechanisms and biomedical applications. Arch Microbiol. 2026;208:99. 10.1007/s00203-025-04618-3. 41493550 10.1007/s00203-025-04618-3

[23] El-Zawawy NA, Abou-Zeid AM, Beltagy DM, Hantera NH, Nouh HS. Mycosynthesis of silver nanoparticles from endophytic *Aspergillus flavipes* AUMC 15772: ovat-statistical optimization, characterization and biological activities. Microb Cell Factories. 2023;22:228. 10.1186/s12934-023-02238-4. 10.1186/s12934-023-02238-4 PMC10629019 37932769

[24] Lakshmeesha TR, Kalagatur NK, Mudili V, Mohan CD, Rangappa S, Prasad BD, et al. Biofabrication of zinc oxide nanoparticles with *Syzygium aromaticum* flower buds extract and finding its novel application in controlling the growth and mycotoxins of *Fusarium graminearum*. Front Microbiol. 2019;10:1244. 10.3389/fmicb.2019.01244. 31249558 10.3389/fmicb.2019.01244 PMC6582371

[25] Caputo F, Mameli M, Sienkiewicz A, Licoccia S, Stellacci F, Ghibelli L, et al. A novel synthetic approach of cerium oxide nanoparticles with improved biomedical activity. Sci Rep. 2017;7:4636. 10.1038/s41598-017-04098-6. 28680107 10.1038/s41598-017-04098-6 PMC5498533

[26] Badawy AME, Luxton TP, Silva RG, Scheckel KG, Suidan MT, Tolaymat TM. Impact of environmental conditions (pH, ionic strength, and electrolyte type) on the surface charge and aggregation of silver nanoparticles suspensions. Environ Sci Technol. 2010;44:1260–6. 10.1021/es902240k. 20099802 10.1021/es902240k

[27] Gaber SE, Hashem AH, El-Sayyad GS, Attia MS. Antifungal activity of myco-synthesized bimetallic ZnO-CuO nanoparticles against fungal plant pathogen *Fusarium oxysporum*. Biomass Convers Bioref. 2024;14:25395–409. 10.1007/s13399-023-04550-w.

[28] Kaur T, Bala M, Kumar G, Vyas A. Biosynthesis of zinc oxide nanoparticles via endophyte *Trichoderma viride* and evaluation of their antimicrobial and antioxidant properties. Arch Microbiol. 2022;204:620. 10.1007/s00203-022-03218-9. 36100763 10.1007/s00203-022-03218-9

[29] Ganesan V, Hariram M, Vivekanandhan S, Muthuramkumar S. *Periconium* sp.(endophytic fungi) extract mediated sol-gel synthesis of ZnO nanoparticles for antimicrobial and antioxidant applications. Mater Sci Semicond Process. 2020;105:104739. 10.1016/j.mssp.2019.104739.

[30] Singh J, Dutta T, Kim K-H, Rawat M, Samddar P, Kumar P. Green’synthesis of metals and their oxide nanoparticles: applications for environmental remediation. J Nanobiotechnol. 2018;16:84. 10.1186/s12951-018-0408-4. 10.1186/s12951-018-0408-4 PMC6206834 30373622

[31] Rai N, Kumari Keshri P, Verma A, Kamble SC, Mishra P, Barik S, et al. Plant associated fungal endophytes as a source of natural bioactive compounds. Mycology. 2021;12:139–59. 10.1080/21501203.2020.1870579. 34552808 10.1080/21501203.2020.1870579 PMC8451683

[32] Bora KS, Sharma A. The genus Artemisia: a comprehensive review. Pharm Biol. 2011;49:101–9. 10.3109/13880209.2010.497815. 20681755 10.3109/13880209.2010.497815

[33] Strobel G, Daisy B. Bioprospecting for microbial endophytes and their natural products. Microbiol Mol Biol Rev. 2003;67:491–502. 10.1128/MMBR.67.4.491-502.2003. 14665674 10.1128/MMBR.67.4.491-502.2003 PMC309047

[34] Shahroudi S, Parvinnasab A, Salahinejad E, Abdi S, Rajabi S, Tayebi L. Efficacy of 3D-printed chitosan–cerium oxide dressings coated with vancomycin-loaded alginate for chronic wounds management. Carbohydr Polym. 2025;349:123036. 10.1016/j.carbpol.2024.123036. 39638529 10.1016/j.carbpol.2024.123036

[35] Manoharan S, Balakrishnan P, Sellappan LK, Sanmugam A. Green synthesized cerium oxide nanoparticles incorporated chitosan-alginate based nanobiopatch for enhanced antibacterial wound dressing applications. J Drug Deliv Sci Technol. 2025;108:106892. 10.1016/j.jddst.2025.106892.

[36] Sun Y, Xia Y. Shape-controlled synthesis of gold and silver nanoparticles. Science. 2002;298:2176–9. 10.1126/science.1077229. 12481134 10.1126/science.1077229

[37] Yang T, Shi Y, Janssen A, Xia Y. Surface capping agents and their roles in shape-controlled synthesis of colloidal metal nanocrystals. Angew Chem Int Ed. 2020;59:15378–401. 10.1002/anie.201911135. 10.1002/anie.201911135 31595609

[38] Fievet F, Lagier J, Figlarz M. Preparing monodisperse metal powders in micrometer and submicrometer sizes by the polyol process. Mrs Bull. 1989;14:29–34. 10.1557/S0883769400060930.

[39] Shaltout KH, Eid EM, Al-Sodany YM, Heneidy SZ, Shaltout SK, El-Masry SA. Effect of protection of mountainous vegetation against over-grazing and over-cutting in South Sinai. Egypt Divers. 2021;13:113. 10.3390/d13030113.

[40] El-Zawawy NA, Ali SS, Khalil MA, Sun J, Nouh HS. Exploring the potential of benzoic acid derived from the endophytic fungus strain *Neurospora crassa* SSN01 as a promising antimicrobial agent in wound healing. Microbiol Res. 2022;262:127108. 10.1016/j.micres.2022.127108. 35797944 10.1016/j.micres.2022.127108

[41] El Zawawy NA, El-Shenody RA, Ali SS, El-Shetehy M. A novel study on the inhibitory effect of marine macroalgal extracts on hyphal growth and biofilm formation of candidemia isolates. Sci Rep. 2020;10:9339. 10.1038/s41598-020-66000-1. 32518329 10.1038/s41598-020-66000-1 PMC7283248

[42] Buchaille L, Freydiere A, Guinet R, Gille Y. Evaluation of six commercial systems for identification of medically important yeasts. Eur J Clin Microbiol Infect Dis. 1998;17:479–88. 10.1007/BF01691130. 9764550 10.1007/BF01691130

[43] Kumar S, Stecher G, Suleski M, Sanderford M, Sharma S, Tamura K. MEGA12: molecular evolutionary genetic analysis version 12 for adaptive and green computing. Mol Biol Evol. 2024;41:msae263. 10.1093/molbev/msae263. 39708372 10.1093/molbev/msae263 PMC11683415

[44] Felsenstein J. Confidence limits on phylogenies: an approach using the bootstrap. Evolution. 1985;39:783–91. 10.1111/j.1558-5646.1985.tb00420.x. 28561359 10.1111/j.1558-5646.1985.tb00420.x

[45] Hanafy BI, Cave GW, Barnett Y, Pierscionek B. Ethylene glycol coated nanoceria protects against oxidative stress in human lens epithelium. RSC Adv. 2019;9:16596–605. 10.1039/C9RA01252D. 35516401 10.1039/c9ra01252d PMC9064421

[46] Allam NG, Shabana SA, Osman YA, Nouh HS. Prevalence of some virulence factors among Gram negative bacteria isolated from patients with lung infection and their antimicrobial susceptibility patterns. Egypt J Bot. 2019;59:633–43. 10.21608/ejbo.2019.6696.1263.

[47] Al-Tohamy R, Ali SS, Saad-Allah K, Fareed M, Ali A, El-Badry A, et al. Phytochemical analysis and assessment of antioxidant and antimicrobial activities of some medicinal plant species from Egyptian flora. J Appl Biomed. 2018;16:289–300. 10.1016/j.jab.2018.08.001.

[48] El Zawawy N, El Shafay S, Abomohra AE-F. Macroalgal activity against fungal urinary tract infections: in vitro screening and evaluation study. Rend Lincei Sci Fis E Nat. 2020;31:165–75. 10.1007/s12210-019-00856-y.

[49] Elshikh M, Ahmed S, Funston S, Dunlop P, McGaw M, Marchant R, et al. Resazurin-based 96-well plate microdilution method for the determination of minimum inhibitory concentration of biosurfactants. Biotechnol Lett. 2016;38:1015–9. 10.1007/s10529-016-2079-2. 26969604 10.1007/s10529-016-2079-2 PMC4853446

[50] Daouk RK, Dagher SM, Sattout EJ. Antifungal activity of the essential oil of *Origanum syriacum* L. J Food Prot. 1995;58:1147–9. 10.4315/0362-028X-58.10.1147. 31137364 10.4315/0362-028X-58.10.1147

[51] Nouh HS, El-Zawawy NA, Halawa M, Shalamesh EM, Ali SS, Korbecka-Glinka G, et al. Endophytic *Penicillium oxalicum* AUMC 14898 from *Opuntia ficus*-indica: a novel source of tannic acid inhibiting virulence and quorum sensing of extensively drug-resistant *Pseudomonas aeruginosa*. Int J Mol Sci. 2024;25:11115. 10.3390/ijms252011115. 39456896 10.3390/ijms252011115 PMC11507641

[52] Christensen GD, Simpson WA, Younger J, Baddour L, Barrett F, Melton D, et al. Adherence of coagulase-negative staphylococci to plastic tissue culture plates: a quantitative model for the adherence of staphylococci to medical devices. J Clin Microbiol. 1985;22:996–1006. 10.1128/jcm.22.6.996-1006.1985. 3905855 10.1128/jcm.22.6.996-1006.1985 PMC271866

[53] Singh S, Patel P, Jaiswal S, Prabhune A, Ramana C, Prasad B. A direct method for the preparation of glycolipid–metal nanoparticle conjugates: sophorolipids as reducing and capping agents for the synthesis of water re-dispersible silver nanoparticles and their antibacterial activity. New J Chem. 2009;33:646–52. 10.1039/B811829A.

[54] Ranjan Sarker S, Polash SA, Boath J, Kandjani AE, Poddar A, Dekiwadia C, et al. Functionalization of elongated tetrahexahedral Au nanoparticles and their antimicrobial activity assay. ACS Appl Mater Interfaces. 2019;11:13450–9. 10.1021/acsami.9b02279. 30869505 10.1021/acsami.9b02279

[55] Zeng X, Tang W, Ye G, Ouyang T, Tian L, Ni Y, et al. Studies on disinfection mechanism of electrolyzed oxidizing water on *E. coli* and *Staphylococcus aureus*. J Food Sci. 2010;75:M253–60. 10.1111/j.1750-3841.2010.01649.x. 20629881 10.1111/j.1750-3841.2010.01649.x

[56] Cho M, Kim J, Kim JY, Yoon J, Kim J-H. Mechanisms of *Escherichia coli* inactivation by several disinfectants. Water Res. 2010;44:3410–8. 10.1016/j.watres.2010.03.017. 20427068 10.1016/j.watres.2010.03.017

[57] Pezeshkpour V, Khosravani SA, Ghaedi M, Dashtian K, Zare F, Sharifi A, et al. Ultrasound assisted extraction of phenolic acids from broccoli vegetable and using sonochemistry for preparation of MOF-5 nanocubes: comparative study based on micro-dilution broth and plate count method for synergism antibacterial effect. Ultrason Sonochem. 2018;40:1031–8. 10.1016/j.ultsonch.2017.09.001. 28946400 10.1016/j.ultsonch.2017.09.001

[58] Chen K, Peng C, Chi F, Yu C, Yang Q, Li Z. Antibacterial and antibiofilm activities of chlorogenic acid against *Yersinia enterocolitica*. Front Microbiol. 2022;13:885092. 10.3389/fmicb.2022.885092. 35602020 10.3389/fmicb.2022.885092 PMC9117966

[59] Liu L, Li J-H, Zi S-F, Liu F-R, Deng C, Ao X, et al. AgNP combined with quorum sensing inhibitor increased the antibiofilm effect on *Pseudomonas aeruginosa*. Appl Microbiol Biotechnol. 2019;103:6195–204. 10.1007/s00253-019-09905-w. 31129741 10.1007/s00253-019-09905-w

[60] Anju V, Busi S, Ranganathan S, Ampasala DR, Kumar S, Suchiang K, et al. Sesamin and sesamolin rescues *Caenorhabditis elegans* from *Pseudomonas aeruginosa* infection through the attenuation of quorum sensing regulated virulence factors. Microb Pathog. 2021;155:104912. 10.1016/j.micpath.2021.104912. 33932548 10.1016/j.micpath.2021.104912

[61] Menaa B, Ribeiro I, Oliveira M, Rahal S, Carvalho MF, Chekireb D. Isolation and characterization of endophytic actinobacteria associated with *Artemisia judaica* L. ssp. sahariensis from desert regions in Algeria. Braz J Microbiol. 2025;56:2347–61. 10.1007/s42770-025-01792-w. 41085894 10.1007/s42770-025-01792-w PMC12660595

[62] Fadiji AE, Mortimer PE, Xu J, Ebenso EE, Babalola OO. Biosynthesis of nanoparticles using endophytes: a novel approach for enhancing plant growth and sustainable agriculture. Sustainability. 2022;14:10839. 10.3390/su141710839.

[63] Dong H, Chen Y-C, Feldmann C. Polyol synthesis of nanoparticles: status and options regarding metals, oxides, chalcogenides, and non-metal elements. Green Chem. 2015;17:4107–32. 10.1039/C5GC00943J.

[64] Hang C, Moawad MS, Lin Z, Guo H, Xiong H, Zhang M, et al. Biosafe cerium oxide nanozymes protect human pluripotent stem cells and cardiomyocytes from oxidative stress. J Nanobiotechnol. 2024;22:132. 10.1186/s12951-024-02383-x. 10.1186/s12951-024-02383-x PMC10967117 38532378

[65] Alpaslan E, Yazici H, Golshan NH, Ziemer KS, Webster TJ. pH-dependent activity of dextran-coated cerium oxide nanoparticles on prohibiting osteosarcoma cell proliferation. ACS Biomater Sci Eng. 2015;1:1096–103. 10.1021/acsbiomaterials.5b00194. 33429551 10.1021/acsbiomaterials.5b00194

[66] Pirmohamed T, Dowding JM, Singh S, Wasserman B, Heckert E, Karakoti AS, et al. Nanoceria exhibit redox state-dependent catalase mimetic activity. Chem Commun. 2010;46:2736–8. 10.1039/b922024k. 10.1039/b922024k PMC3038687 20369166

[67] De Marzi L, Monaco A, De Lapuente J, Ramos D, Borras M, Di Gioacchino M, et al. Cytotoxicity and genotoxicity of ceria nanoparticles on different cell lines in vitro. Int J Mol Sci. 2013;14:3065–77. 10.3390/ijms14023065. 23377016 10.3390/ijms14023065 PMC3588031

[68] Coates J. Interpretation of infrared spectra, a practical approach. Encycl Anal Chem. 2000;12:10815–37. 10.1002/9780470027318.a5606.

[69] Korsvik C, Patil S, Seal S, Self WT Superoxide dismutase mimetic properties exhibited by vacancy engineered ceria nanoparticles. Chem Commun. 2007. 10.1039/b615134e. 10.1039/b615134e 17325804

[70] Shao X, Wei X, Song X, Hao L, Cai X, Zhang Z, et al. Independent effect of polymeric nanoparticle zeta potential/surface charge, on their cytotoxicity and affinity to cells. Cell Prolif. 2015;48:465–74. 10.1111/cpr.12192. 26017818 10.1111/cpr.12192 PMC6496505

[71] Suk JS, Lai SK, Boylan NJ, Dawson MR, Boyle MP, Hanes J. Rapid transport of muco-inert nanoparticles in cystic fibrosis sputum treated with N-acetyl cysteine. Nanomed. 2011;6:365–75. 10.2217/nnm.10.123. 10.2217/nnm.10.123 PMC3102009 21385138

[72] Torge A, Wagner S, Chaves PS, Oliveira EG, Guterres SS, Pohlmann AR, et al. Ciprofloxacin-loaded lipid-core nanocapsules as mucus penetrating drug delivery system intended for the treatment of bacterial infections in cystic fibrosis. Int J Pharm. 2017;527:92–102. 10.1016/j.ijpharm.2017.05.013. 28499793 10.1016/j.ijpharm.2017.05.013

[73] Peng D, Huang Z, Zhang X. Breath and life: emerging nanotechnologies for cystic fibrosis therapy. Fibrosis. 2025;3:10014. 10.70322/fibrosis.2025.10014.

[74] Farias IAP, dos Santos CCL, Sampaio FC. Antimicrobial activity of cerium oxide nanoparticles on opportunistic microorganisms: a systematic review. BioMed Res Int. 2018;2018:1923606. 10.1155/2018/1923606. 29607315 10.1155/2018/1923606 PMC5827881

[75] Qi M, Li W, Zheng X, Li X, Sun Y, Wang Y, et al. Cerium and its oxidant-based nanomaterials for antibacterial applications: a state-of-the-art review. Front Mater. 2020;7:213. 10.3389/fmats.2020.00213.

[76] Akhil K, Jayakumar J, Gayathri G, Khan SS. Effect of various capping agents on photocatalytic, antibacterial and antibiofilm activities of ZnO nanoparticles. J Photochem Photobiol B. 2016;160:32–42. 10.1016/j.jphotobiol.2016.03.015. 27088507 10.1016/j.jphotobiol.2016.03.015

[77] Kannan S, Sundrarajan M. A green approach for the synthesis of a cerium oxide nanoparticle: characterization and antibacterial activity. Int J Nanosci. 2014;13:1450018. 10.1142/S0219581X14500185.

[78] Arumugam A, Karthikeyan C, Hameed ASH, Gopinath K, Gowri S, Karthika V. Synthesis of cerium oxide nanoparticles using *Gloriosa superba* L. leaf extract and their structural, optical and antibacterial properties. Mater Sci Eng C. 2015;49:408–15. 10.1016/j.msec.2015.01.042. 10.1016/j.msec.2015.01.042 25686966

[79] Reddy Yadav L, Manjunath K, Archana B, Madhu C, Raja Naika H, Nagabhushana H, et al. Fruit juice extract mediated synthesis of CeO2 nanoparticles for antibacterial and photocatalytic activities. Eur Phys J Plus. 2016;131:154. 10.1140/epjp/i2016-16154-y.

[80] Zhang M, Zhang C, Zhai X, Luo F, Du Y, Yan C, 氧化铈纳米粒子的抗菌机理及应用. Sci China Mater. 2019;62:1727–39. 10.1007/s40843-019-9471-7.

[81] Pop OL, Mesaros A, Vodnar DC, Suharoschi R, Tăbăran F, Magerușan L, et al. Cerium oxide nanoparticles and their efficient antibacterial application in vitro against gram-positive and gram-negative pathogens. Nanomaterials. 2020;10:1614. 10.3390/nano10081614. 32824660 10.3390/nano10081614 PMC7466638

[82] Arasu MV, Thirumamagal R, Srinivasan M, Al-Dhabi NA, Ayeshamariam A, Kumar DS, et al. Green chemical approach towards the synthesis of CeO2 doped with seashell and its bacterial applications intermediated with fruit extracts. J Photochem Photobiol B. 2017;173:50–60. 10.1016/j.jphotobiol.2017.05.032. 28564630 10.1016/j.jphotobiol.2017.05.032

[83] Aliashkevich A, Alvarez L, Cava F. New insights into the mechanisms and biological roles of D-amino acids in complex eco-systems. Front Microbiol. 2018;9:683. 10.3389/fmicb.2018.00683. 29681896 10.3389/fmicb.2018.00683 PMC5898190

[84] Imlay JA. Cellular defenses against superoxide and hydrogen peroxide. Annu Rev Biochem. 2008;77:755–76. 10.1146/annurev.biochem.77.061606.161055. 18173371 10.1146/annurev.biochem.77.061606.161055 PMC3057177

[85] Shao T, Yang G, Wang M, Lu Z, Min H, Zhao L. Reduction of oxidative stress by bioaugmented strain Pseudomonas sp. HF-1 and selection of potential biomarkers in sequencing batch reactor treating tobacco wastewater. Ecotoxicology. 2010;19:1117–23. 10.1007/s10646-010-0494-z. 20396945 10.1007/s10646-010-0494-z

[86] Kim JS, Kuk E, Yu KN, Kim J-H, Park SJ, Lee HJ, et al. Antimicrobial effects of silver nanoparticles. Nanomed Nanotechnol Biol Med. 2007;3:95–101. 10.1016/j.nano.2006.12.001. 10.1016/j.nano.2006.12.001 17379174

[87] Chernousova S, Epple M. Silver as antibacterial agent: ion, nanoparticle, and metal. Angew Chem Int Ed. 2013;52:1636–53. 10.1002/anie.201205923. 10.1002/anie.201205923 23255416

[88] Carlson C, Hussain SM, Schrand AM, Braydich-Stolle K, Hess L, Jones KL. Unique cellular interaction of silver nanoparticles: size-dependent generation of reactive oxygen species. J Phys Chem B. 2008;112:13608–19. 10.1021/jp712087m. 18831567 10.1021/jp712087m

[89] Ramalingam B, Parandhaman T, Das SK. Antibacterial effects of biosynthesized silver nanoparticles on surface ultrastructure and nanomechanical properties of gram-negative bacteria viz. *Escherichia coli* and *Pseudomonas aeruginosa*. ACS Appl Mater Interfaces. 2016;8:4963–76. 10.1021/acsami.6b00161. 26829373 10.1021/acsami.6b00161

[90] Du T, Chen S, Zhang J, Li T, Li P, Liu J, et al. Antibacterial activity of manganese dioxide nanosheets by ROS-mediated pathways and destroying membrane integrity. Nanomaterials. 2020;10:1545. 10.3390/nano10081545. 32784527 10.3390/nano10081545 PMC7466589

[91] Flemming H-C, Wingender J. The biofilm matrix. Nat Rev Microbiol Nat. 2010;8:623–33. 10.1038/nrmicro2415. 10.1038/nrmicro2415 20676145

[92] Al-Shabib NA, Husain FM, Hassan I, Khan MS, Ahmed F, Qais FA, et al. Biofabrication of zinc oxide nanoparticle from *Ochradenus baccatus* leaves: broad-spectrum antibiofilm activity, protein binding studies, and in vivo toxicity and stress studies. J Nanomater. 2018;2018:8612158. 10.1155/2018/8612158.

[93] Al-Shabib NA, Husain FM, Ahmad N, Qais FA, Khan A, Khan A, et al. Facile synthesis of tin oxide hollow nanoflowers interfering with quorum sensing-regulated functions and bacterial biofilms. J Nanomater. 2018;2018:6845026. 10.1155/2018/6845026.

[94] Qais FA, Shafiq A, Ahmad I, Husain FM, Khan RA, Hassan I. Green synthesis of silver nanoparticles using *Carum copticum*: assessment of its quorum sensing and biofilm inhibitory potential against gram negative bacterial pathogens. Microb Pathog. 2020;144:104172. 10.1016/j.micpath.2020.104172. 32224208 10.1016/j.micpath.2020.104172

[95] Altaf M, Parveen N, Qais FA, Abdullah K, Ahmad I. Control of biofilm and virulence in *Pseudomonas aeruginosa* by green-synthesized titanium–cerium nanocomposites. Microbiol Res. 2023;14:1653–69. 10.3390/microbiolres14040114.

[96] Roy R, Tiwari M, Donelli G, Tiwari V. Strategies for combating bacterial biofilms: a focus on anti-biofilm agents and their mechanisms of action. Virulence. 2018;9:522–54. 10.1080/21505594.2017.1313372. 28362216 10.1080/21505594.2017.1313372 PMC5955472

[97] Rabin N, Zheng Y, Opoku-Temeng C, Du Y, Bonsu E, Sintim HO. Biofilm formation mechanisms and targets for developing antibiofilm agents. Future Med Chem. 2015;7:493–512. 10.4155/fmc.15.6. 25875875 10.4155/fmc.15.6

[98] O’Toole G, Kaplan HB, Kolter R. Biofilm formation as microbial development. Annu Rev Microbiol. 2000;54:49–79. 10.1146/annurev.micro.54.1.49. 11018124 10.1146/annurev.micro.54.1.49

[99] Lewandowski Z Fundamentals of biofilm research. CRC; 2013. 10.1201/b16291.

[100] Li Y, Xiao P, Wang Y, Hao Y. Mechanisms and control measures of mature biofilm resistance to antimicrobial agents in the clinical context. ACS Omega. 2020;5:22684–90. 10.1021/acsomega.0c02294. 32954115 10.1021/acsomega.0c02294 PMC7495453

[101] Karygianni L, Ren Z, Koo H, Thurnheer T. Biofilm matrixome: extracellular components in structured microbial communities. Trends Microbiol. 2020;28:668–81. 10.1016/j.tim.2020.03.016. 32663461 10.1016/j.tim.2020.03.016

[102] Qayyum S, Khan AU. Nanoparticles vs. biofilms: a battle against another paradigm of antibiotic resistance. MedChemComm. 2016;7:1479–98. 10.1039/C6MD00124F.

[103] Paluch E, Rewak-Soroczyńska J, Jędrusik I, Mazurkiewicz E, Jermakow K. Prevention of biofilm formation by quorum quenching. Appl Microbiol Biotechnol. 2020;104:1871–81. 10.1007/s00253-020-10349-w. 31927762 10.1007/s00253-020-10349-w PMC7007913

[104] Mishra R, Kushveer JS, Khan MIK, Pagal S, Meena CK, Murali A, et al. 2, 4-Di-tert-butylphenol isolated from an endophytic fungus, *Daldinia eschscholtzii*, reduces virulence and quorum sensing in *Pseudomonas aeruginosa*. Front Microbiol. 2020;11:1668. 10.3389/fmicb.2020.01668. 32849344 10.3389/fmicb.2020.01668 PMC7418596

[105] Fong J, Mortensen KT, Nørskov A, Qvortrup K, Yang L, Tan CH, et al. Itaconimides as novel quorum sensing inhibitors of *Pseudomonas aeruginosa*. Front Cell Infect Microbiol. 2019;8:443. 10.3389/fcimb.2018.00443. 30666301 10.3389/fcimb.2018.00443 PMC6330316

[106] Franklin MJ, Nivens DE, Weadge JT, Howell PL. Biosynthesis of the *Pseudomonas aeruginosa* extracellular polysaccharides, alginate, Pel, and Psl. Front Microbiol. 2011;2:167. 10.3389/fmicb.2011.00167. 21991261 10.3389/fmicb.2011.00167 PMC3159412

[107] Bomberger JM, MacEachran DP, Coutermarsh BA, Ye S, O’Toole GA, Stanton BA. Long-distance delivery of bacterial virulence factors by *Pseudomonas aeruginosa* outer membrane vesicles. PLoS Pathog. 2009;5:e1000382. 10.1371/journal.ppat.1000382. 19360133 10.1371/journal.ppat.1000382 PMC2661024

[108] Vasil ML. Pseudomonas aeruginosa phospholipases and phospholipids. *Pseudomonas*. Mol Biol Emerg Issues. 2006;4:69–97. 10.1007/0-387-28881-3_3

[109] Meyers DJ, Palmer KC, Bale LA, Kernacki K, Preston M, Brown T, et al. In vivo and in vitro toxicity of phospholipase C from *Pseudomonas aeruginosa*. Toxicon. 1992;30:161–9. 10.1016/0041-0101(92)90469-L. 1557786 10.1016/0041-0101(92)90469-l

[110] Wargo MJ, Ho TC, Gross MJ, Whittaker LA, Hogan DA. GbdR regulates *Pseudomonas aeruginosa* plcH and pchP transcription in response to choline catabolites. Infect Immun. 2009;77:1103–11. 10.1128/IAI.01008-08. 19103776 10.1128/IAI.01008-08 PMC2643652

[111] Kessler E, Safrin M, Olson JC, Ohman D. Secreted LasA of *Pseudomonas aeruginosa* is a staphylolytic protease. J Biol Chem. 1993;268:7503–8. 10.1016/S0021-9258(18)53203-8. 8463280

[112] Musthafa KS, Saroja V, Pandian SK, Ravi AV. Antipathogenic potential of marine *Bacillus* sp. SS4 on *N*-acyl-homoserine-lactone-mediated virulence factors production in *Pseudomonas aeruginosa* (PAO1). J Biosci. 2011;36:55–67. 10.1007/s12038-011-9011-7. 21451248 10.1007/s12038-011-9011-7

[113] Abdelraheem WM, Mohamed ES. The effect of Zinc Oxide nanoparticles on *Pseudomonas aeruginosa* biofilm formation and virulence genes expression. J Infect Dev Ctries. 2021;15:826–32. 10.3855/jidc.13958. 34242193 10.3855/jidc.13958

