# Supplementary material for: Harnessing the endophytic Clavispora lusitaniae for the biogenic synthesis of ethylene glycol-capped cerium oxide nanoparticles: a green approach to combat respiratory Pseudomonas aeruginosa
Source: Microb Cell Fact. 2026 Aug 20;25:177. doi: 10.1186/s12934-026-03091-x (PMC13495233; doi:10.1186/s12934-026-03091-x)
Supplement: Supplementary file 1 [file 12934_2026_3091_MOESM1_ESM.docx]

**Supplementary file**

**Harnessing the endophytic *Clavispora lusitaniae* for the biogenic synthesis of ethylene glycol-capped cerium oxide nanoparticles: A green approach to combat respiratory *Pseudomonas aeruginosa***

**Hoda S. Nouh^1^, Nessma A. El-Zawawy^1^, Shimaa El-Sapagh^1^, Ebrahim M. shalamesh^1,2^, Sally M. Metwally^1^**

**Table S1. Primers of *P. aeruginosa* used in qRT-PCR study**

|  | Gene | Forward primer (5'-3') | Reverse primer (5'-3') | Reference |
| --- | --- | --- | --- | --- |
| 1 | *proC* (Reference gene) | CAGGCCGGGCAGTTGCTGTC | GGTCAGGCGCGAGGCTGTCT | [63] |
| 2 | *lasR* | ACGCTCAAGTGGAAAATTGG | TCGTAGTCCTGGCTGTCCTT | [63] |
| 3 | *lasI* | GGCTGGGACGTTAGTGTCAT | AAAACCTGGGCTTCAGGAGT | [63] |
| 4 | *rhlI* | AAGGACGTCTTCGCCTACCT | GCAGGCTGGACCAGAATATC | [63] |
| 5 | *rhlR* | CATCCGATGCTGATGTCCAACC | ATGATGGCGATTTCCCCGGAC | [63] |
| 6 | *lasB* | GACCGAGAATGACAAAGTGGAA | GGTAGGAGACGTTGTAGACCGT | [63] |
| 7 | *aprA* | CCCTGTCCTATTCGTTCCTG | GCGTCGACGAAGTGGATATT | [63] |
| 8 | *toxA* | GGAGCGCAACTATCCCACT | TGGTAGCCGACGAACACATA | [63] |
| 9 | *exoS* | GGCGGATGCGGAAAAGTAC | CTGACGCAGAGCGCGATT | [63] |
| 10 | *pelF* | GCCGATGAAGAACACCGAGAAGG | CCGATGTCTGCCTGCTGCTG | [63] |
| 11 | *pslA* | GCCGCTTCATCCGCAAGACC | TGTATTCGCTGACCGCCTCCTC | [63] |
| 12 | *pqsR* | CTGATCTGCCGGTAATTGG | ATCGACGAGGAACTGAAGA | [63] |
| 13 | *pclH* | ACCTGGTGGATAGGGCTCGCCG | GTGCTGACCGTGGAAAGGCCGA | [63] |
| 14 | *algD* | GCGACCTGGACCTGGGCT | TCCTCGATCAGCGGGATC | [63] |
| 15 | *gacA* | GTGATTAAGGTGCGTGGTGGTC | CGGTAGCGATAGGTATTCACG | [63] |

|  |  |  |
| --- | --- | --- |

**Fig. S1 Fresh and preserved *Artemisia judaica* collected from Salouga and Ghazal Nature Reserve at Egypt**

**Table S2. Consolidated Physicochemical and Drug-Likeness Properties of Ethylene Glycol (EG)**

| **Property Category** | **Parameter** | **Value/Result** |
| --- | --- | --- |
| **Identity** | Chemical Formula | C₂H₆O₂ |
|  | Canonical SMILES | C(CO)O |
| **Physical Properties** | Molecular Weight | **62.07 g/mol** |
|  | Topological Polar Surface Area (TPSA) | **40.46 Å²** |
|  | Fraction Csp3 | 1.00 |
|  | Num. Rotatable Bonds | 1 |
| **Lipophilicity** | Consensus Log *P* | -0.70 |
| **Solubility** | Water Solubility Class (ESOL/Ali) | Highly soluble (Log S: 0.70 / 1.01) |
| **Drug-Likeness** | H-bond Donors | 2 |
|  | H-bond Acceptors | 2 |
|  | Lipinski Violations | 0 |
|  | Bioavailability Score | 0.55 |

**Table S3. Consolidated ADMET and Toxicity Profile of Ethylene Glycol (EG)**

| **ADMET Feature** | **Endpoint/Target** | **Prediction Status** | **Probability/Score** |
| --- | --- | --- | --- |
| **Absorption** | GI Absorption | High | N/A (Qualitative Prediction) |
|  | Skin Permeation (log *Kp*) | N/A | -7.64 cm/s |
|  | BBB Permeant (SwissADME) | No | N/A (Qualitative Prediction) |
| **Distribution** | P-gp Substrate | No | N/A (Qualitative Prediction) |
| **Metabolism** | CYP1A2 Inhibitor | No | N/A (Qualitative Prediction) |
|  | CYP2C19 Inhibitor | No | N/A (Qualitative Prediction) |
|  | CYP2C9 Inhibitor | No | N/A (Qualitative Prediction) |
|  | CYP2D6 Inhibitor | No | N/A (Qualitative Prediction) |
|  | CYP3A4 Inhibitor | No | N/A (Qualitative Prediction) |
| **Organ Toxicity** | Hepatotoxicity | Inactive | 0.98 |
|  | Neurotoxicity | Inactive | 0.84 |
|  | Nephrotoxicity | Inactive | 0.55 |
|  | Cardiotoxicity | Active | 0.80 |
|  | Respiratory Toxicity | Inactive | 0.79 |
| **Toxicity Endpoints** | Mutagenicity | Inactive | 0.94 |
|  | Immunotoxicity | Inactive | 0.99 |
|  | Carcinogenicity | Inactive | 0.86 |
|  | Cytotoxicity | Inactive | 0.82 |
|  | BBB-barrier¹ | Active | 0.54 |

¹ **Note on Blood-Brain Barrier (BBB) Predictions:** The "No" prediction from SwissADME refers specifically to the absence of passive membrane permeability. In contrast, the "Active" status (0.54) predicted by ProTox-III refers to a toxicological endpoint or potential interaction with the barrier's active transport/integrity mechanisms.

**Methodological Notes for Supplementary Tables**

**Computational Platforms:** Physicochemical and pharmacokinetic properties (including GI absorption, BBB permeation, and skin permeation) were predicted using the SwissADME web server. Toxicity endpoints and organ toxicity predictions were generated using the ProTox-III platform.

**Note on Interpretation:** These in silico predictions apply exclusively to the free Ethylene Glycol molecule. They do not account for the biological behavior, altered surface chemistry, or pharmacokinetic profile of the complex EG-CeO₂NP nanostructure.

**Nephrotoxicity Caveat:** While the predicted status for nephrotoxicity is "Inactive," the probability score (0.55) resides near the baseline of random chance (0.50), indicating low computational confidence. This is critical as EG systemically metabolizes into oxalic acid, a known inducer of acute kidney injury.

**Confidence in Toxicity Predictions:** For ProTox-III results, probability scores range from 0 to 1. Scores closer to 1.0 indicate higher machine-learning confidence. Scores between 0.50 and 0.60 (as seen with Nephrotoxicity and BBB-barrier) signify "Low Confidence" results that require experimental validation.

**Table S9: Statistical Summary Report: Growth Inhibition of EG-CeO2NPs against *P. aeruginosa* (PA-5)**

| **Dunnett's multiple comparisons test** | **Below threshold?** | Summary | Adjusted P Value |  |  |
| --- | --- | --- | --- | --- | --- |
|  |  |  |  |  |  |
| **0 hr** |  |  |  |  |  |
| Control vs. 1/2MIC | No | ns | >0.9999 |  |  |
| Control vs. 1/4MIC | No | ns | >0.9999 |  |  |
| Control vs. 1/8MIC | No | ns | >0.9999 |  |  |
| Control vs. 1/16MIC | No | ns | >0.9999 |  |  |
|  |  |  |  |  |  |
| **2 hr** |  |  |  |  |  |
| Control vs. 1/2MIC | Yes | ** | 0.0088 |  |  |
| Control vs. 1/4MIC | Yes | ** | 0.0088 |  |  |
| Control vs. 1/8MIC | Yes | ** | 0.0088 |  |  |
| Control vs. 1/16MIC | No | ns | 0.9687 |  |  |
|  |  |  |  |  |  |
| **4 hr** |  |  |  |  |  |
| Control vs. 1/2MIC | Yes | ** | 0.0088 |  |  |
| Control vs. 1/4MIC | No | ns | 0.0609 |  |  |
| Control vs. 1/8MIC | No | ns | 0.7364 |  |  |
| Control vs. 1/16MIC | No | ns | 0.8810 |  |  |
|  |  |  |  |  |  |
| **6 hr** |  |  |  |  |  |
| Control vs. 1/2MIC | Yes | **** | <0.0001 |  |  |
| Control vs. 1/4MIC | Yes | **** | <0.0001 |  |  |
| Control vs. 1/8MIC | Yes | *** | 0.0002 |  |  |
| Control vs. 1/16MIC | No | ns | 0.7364 |  |  |
|  |  |  |  |  |  |
| **8 hr** |  |  |  |  |  |
| Control vs. 1/2MIC | Yes | **** | <0.0001 |  |  |
| Control vs. 1/4MIC | Yes | **** | <0.0001 |  |  |
| Control vs. 1/8MIC | No | ns | 0.1058 |  |  |
| Control vs. 1/16MIC | No | ns | 0.8810 |  |  |
|  |  |  |  |  |  |
| **10 hr** |  |  |  |  |  |
| Control vs. 1/2MIC | Yes | **** | <0.0001 |  |  |
| Control vs. 1/4MIC | Yes | **** | <0.0001 |  |  |
| Control vs. 1/8MIC | Yes | **** | <0.0001 |  |  |
| Control vs. 1/16MIC | Yes | **** | <0.0001 |  |  |
|  |  |  |  |  |  |
| **12 hr** |  |  |  |  |  |
| Control vs. 1/2MIC | Yes | **** | <0.0001 |  |  |
| Control vs. 1/4MIC | Yes | **** | <0.0001 |  |  |
| Control vs. 1/8MIC | Yes | **** | <0.0001 |  |  |
| Control vs. 1/16MIC | No | ns | 0.0609 |  |  |
|  |  |  |  |  |  |
| **24 hr** |  |  |  |  |  |
| Control vs. 1/2MIC | Yes | **** | <0.0001 |  |  |
| Control vs. 1/4MIC | Yes | **** | <0.0001 |  |  |
| Control vs. 1/8MIC | Yes | **** | <0.0001 |  |  |
| Control vs. 1/16MIC | No | ns | 0.0609 |  |  |
|  |  |  |  |  |  |

**Note: **** : *P*<0.0001; *** : *P*<0.001, * : *P*<0.05; ns : *P*>0.05 (No Significance).**

**Table S10: Statistical Summary Report: Growth Inhibition of EG-CeO2NPs against *P. aeruginosa* (PA-ATCC27853)**

| **Dunnett's multiple comparisons test** | **Below threshold?** | **Summary** | **Adjusted P Value** |
| --- | --- | --- | --- |
| **0 hr** |  |  |  |
| Control vs. 1/2MIC | No | ns | >0.9999 |
| Control vs. 1/4MIC | No | ns | >0.9999 |
| Control vs. 1/8MIC | No | ns | >0.9999 |
| Control vs. 1/16MIC | No | ns | >0.9999 |
| **2hr** |  |  |  |
| Control vs. 1/2MIC | Yes | *** | 0.0007 |
| Control vs. 1/4MIC | Yes | *** | 0.0007 |
| Control vs. 1/8MIC | Yes | *** | 0.0007 |
| Control vs. 1/16MIC | No | ns | 0.9958 |
| **4hr** |  |  |  |
| Control vs. 1/2MIC | Yes | *** | 0.0007 |
| Control vs. 1/4MIC | No | ns | 0.1657 |
| Control vs. 1/8MIC | No | ns | 0.6293 |
| Control vs. 1/16MIC | No | ns | 0.9480 |
| **6hr** |  |  |  |
| Control vs. 1/2MIC | Yes | **** | <0.0001 |
| Control vs. 1/4MIC | Yes | * | 0.0230 |
| Control vs. 1/8MIC | No | ns | 0.4378 |
| Control vs. 1/16MIC | Yes | * | 0.0473 |
| **8hr** |  |  |  |
| Control vs. 1/2MIC | Yes | **** | <0.0001 |
| Control vs. 1/4MIC | Yes | *** | 0.0007 |
| Control vs. 1/8MIC | No | ns | >0.9999 |
| Control vs. 1/16MIC | No | ns | 0.8175 |
| **10hr** |  |  |  |
| Control vs. 1/2MIC | Yes | **** | <0.0001 |
| Control vs. 1/4MIC | Yes | **** | <0.0001 |
| Control vs. 1/8MIC | Yes | * | 0.0230 |
| Control vs. 1/16MIC | Yes | **** | <0.0001 |
| **12hr** |  |  |  |
| Control vs. 1/2MIC | Yes | **** | <0.0001 |
| Control vs. 1/4MIC | Yes | **** | <0.0001 |
| Control vs. 1/8MIC | Yes | * | 0.0230 |
| Control vs. 1/16MIC | Yes | **** | <0.0001 |
| **24hr** |  |  |  |
| Control vs. 1/2MIC | Yes | **** | <0.0001 |
| Control vs. 1/4MIC | Yes | **** | <0.0001 |
| Control vs. 1/8MIC | Yes | * | 0.0230 |
| Control vs. 1/16MIC | No | ns | >0.9999 |

**Note: **** : *P*<0.0001; *** : *P*<0.001, * : *P*<0.05; ns : *P*>0.05 (No Significance).**

**Table S11. RT-PCR analysis of virulence genes expression in *P. aeruginosa* PA-5 in the presence of EG-CeO_2_ NPs.**

| Gene | Description | Relative Gene Expression (Fold Change) | Reduction in gene expression (% ) | *p*- value |
| --- | --- | --- | --- | --- |
| *pelF* | PelF | 0.52 | 48 | <0.0001 |
| *pslA* | PslA | 0.49 | 51 | <0.0001 |
| *algD* | GDP-mannose 6-dehydrogenase AlgD | 0.47 | 53 | <0.0001 |
| *gacA* | Response regulator GacA | 0.48 | 52 | <0.0001 |
| *rhlR* | Transcriptional regulator RhlR | 0.75 | 25 | 0.0336 |
| *lasR* | Transcriptional regulator LasR | 0.43 | 57 | <0.0001 |
| *pqsR* | Transcriptional regulator MvfR | 0.21 | 79 | <0.0001 |
| *rhlI* | Autoinducer synthesis protein RhlI | 0.62 | 38 | 0.0005 |
| *lasI* | Autoinducer synthesis protein LasI | 0.42 | 58 | <0.0001 |
| *lasB* | Elastase LasB | 0.22 | 78 | <0.0001 |
| *plcH* | Hemolytic phospholipase C precursor | 0.31 | 69 | <0.0001 |
| *toxA* | Exotoxin A precursor | 0.15 | 85 | <0.0001 |
| *exoS* | Exoenzyme S | 0.13 | 87 | <0.0001 |
